# Losers and losses of COVID-19: a directed technical change analysis with fiscal and monetary policies

**DOI:** 10.1007/s10644-023-09486-9

**Published:** 2023-03-02

**Authors:** Oscar Afonso

**Affiliations:** Faculdade of Economics, and CEFUP, University of Porto, Rua Dr. Roberto Frias, 4200-464 Porto, Portugal

**Keywords:** Public and private sectors, Fiscal and monetary policies, Technological-knowledge gap, Wage inequality, Numerical computations, O31, O33, O38, J31, C63, E52, E62

## Abstract

We develop a directed technical change growth model with both public and private sectors. Due to the COVID-19 pandemic, labor productivity and R &D activity in the private sector are considered to have a negative shock. The former shock causes an immediate fall in the private premium, which can be reversed during transition dynamics towards the steady-state. Fiscal policies are materialized in direct and indirect R &D subsidies, and the monetary policies consist of relaxing cash-in-advance restrictions. An appropriate fiscal policy, together or not with monetary policy, can restore the pre-shock situation. Monetary policy is reinforced in the presence of monetary-transaction costs on consumption and of money-in-the-utility function.

## Introduction

The COVID-19 pandemic has claimed thousands of lives on every continent and, for a time, crippled the world economy. Due to governments’ initial guidance to stay home—more than a third of the global population was placed in confinement (Kaplan et al. 2020)—activities involving groups of people were momentarily suspended and, excluding purchases of products such as food, drinks, cleaning products, and medicines, the remaining purchases were postponed. On the uncertainty side, demand has retreated because people have chosen to save even because of the risk of job loss, and on the supply side, industrial production has slowed even because it requires face-to-face activity and the number of patients and people at home has increased. Changes in consumption patterns, quarantine, supply constraints and the disruption of international value chains have led to disruptions in business activities in many (private) economic sectors.^1^ In short, the economy cooled, many private businesses closed, and progressively less money started circulating.

Focusing the analysis on advanced economies, the private sector lost out in relative terms against the public sector because it operates primarily in activities that require physical presence (Buchanan et al. 2020) and this distinction serves as the motivation for the paper.^2^ Differences in effects between the public and private sectors can undermine the economic and social cohesion and economic growth that underpins society’s standard of living.

In this context, the fall in economic activity was initially an inevitable consequence of the increase in the number of patients and the government measures imposed to limit the spread of the disease. At the same time, the government was forced to impose measures to support economic activity by preserving the productive (private) sector, ensuring the possible functioning of the production machine, and avoiding excessive economic disturbances, with the intention of abolishing them upon the return to normalcy. In this process of supporting the economy, fiscal and monetary stimulus was the appropriate response in most advanced economies. A successful recovery depends on public health actions to control the virus, and also depends on policy measures by governments and central banks to stimulate income recovery for the most affected households and businesses. Such measures should ensure that businesses, jobs, and overall economic and trade networks are maintained to facilitate rapid recovery.

Keeping in mind the context of the COVID-19 pandemic, the paper aims to analyze the impact of the crisis on the following key macroeconomic variables: relative importance of the public and private sectors, progress and direction of technological knowledge, wage inequality between public and private sector workers, growth rate of the economy, inflation rate of the economy, and social welfare in each of the sectors. To address the differential impact of the crisis on the public and private sectors and the effects of fiscal and monetary policies on recovery, we follow, extending it, the mechanisms of the Direct Technical Change (DTC) framework—e.g., Acemoglu and Zilibotti (2001), Acemoglu (2002), and Afonso (2006). Extensions to the baseline DTC model to meet the objectives contribute to several existing guidelines in the literature; in particular to the literature that examines: (i) the channels through which the current pandemic crisis may affect workers differently (e.g., Alon et al. 2020; Dingel and Neiman 2020; Mongey and Weinberg 2020; ii) the impact of economic crises on the labor market (e.g., Hoynes et al. 2012; Christiano et al. 2015; iii) the importance of policy measures to dampen economic shocks (e.g., Hoynes et al. 2012, Giupponi and Landais 2018, Cahuc et al. 2018; Kopp and Siegenthaler 2018).

To further detail the methodology followed, it should be noted that, particularly since the 1980s, monetary policy has been the responsibility of central banks. Moreover, fiscal and monetary policies were constrained by binding rules aimed at avoiding excessive public deficits in the case of fiscal policy and excessive money supply in the case of monetary policy. As stated by Afonso and Forte (2021), the need for fiscal and monetary discipline was justified by the potential negative external effects resulting from excessive deficits and due to unwanted inflation. For example, in the European Monetary Union, fiscal and monetary rules were aimed at maintaining price stability to ensure external competitiveness (Baldwin and Wyplosz 2004). The need for discipline has been further reinforced by the Stability and Growth Pact rules that take, as a rule, a balanced government budget as a key medium-term objective.

Fiscal discipline is therefore intended to avoid a possible increase in interest rates, avoiding pressure on central banks to implement a more expansionary monetary policy and, consequently, an increase in inflation (De Grauwe 2007), which, according to the existing theoretical and empirical literature, penalizes economic growth (e.g., Benhabib and Spiegel 2009; Afonso and Forte 2021). In any case, some public spending on R &D was encouraged (Afonso and Alves 2009; Afonso et al. 2009). In line with this procedure, to promote economic recovery, it is considered that governments can directly provide R &D subsidies by encouraging the discovery of new prototypes/designs, and they can indirectly support R &D by subsidizing the production of intermediate goods that materialize the economic use of the prototypes (Cozzi and Impullitti 2010).

In the case of monetary policy, central banks have two main instruments at their disposal, which, differently, are based on increasing the amount of money in circulation in economies. The first option is to issue money, but this has little effect in the long run and causes inflation. The second option is to reduce the interest rate, stimulating credit and, consequently, consumption and investment. In general, monetary policy materializes in steering the nominal interest rate and, in this way, affects the economy—a viewpoint followed in this paper. The choice of modeling raises the question of whether liquidity matters for output in the real economy. Now the literature provides ample evidence that production requires money (Opler et al. 1999; Mikkelson and Partch 2003; Bates et al. 2009; McLean 2011). The literature that has recently introduced the demand for money into endogenous growth theory considers that money affects the economy because individuals like to have money and therefore include money in their utility function and/or money affects the economy because producers need to make payments, including cash-in-advance (CIA) constraints—Stockman (1981) and Wang and Yip (1992). In the latter case, a CIA constraint on R &D investment is generally considered (Chu and Cozzi 2014; Gil and Iglésias 2019; Afonso and Pinho 2022), since there is a strong sensitivity to R &D cash flow in firms (Brown and Petersen 2009, 2011, 2015; Brown et al. 2009, 2012; Falato and Sim 2014; Chu et al. 2017; Afonso and Pinho 2022). The effects in both cases considered by the literature—money affects the economy as individuals like to hold cash or producers/entrepreneurs need cash to make payments—are generally very similar. Thus, two different CIA constraints are considered: one faced by intermediate goods producers and the other by prototype producers in the R &D sector.

Following a change in the nominal interest rate and different CIAs—between the private and public sectors, and between the intermediate goods and R &D subsectors—a force is generated that transmits inflation costs that distort incentives and the use of economic resources. In fact, private firms are considered to have greater liquidity needs and therefore need to borrow more expensive money for a given amount of investment or production. To our knowledge, there are no empirical studies that explicitly analyze the relationship between the incidence of private or public firms and their liquidity needs. However, since the government serves to ensure the fulfillment of responsibilities in the public sector, the CIA associated with the public sector is expected to be less demanding.

In short, considering, (i) the abrupt fall in economic activity, expressed in the destruction of the productive capacity of the private sector in relative terms and, for us, materialized in the reduction of the respective labor productivity, and (ii) recovery driven by an increase in resources devoted to private R &D activities in the face of public intervention with subsidies and less stringent CIA constraints, we intend to analyze the effect of different packages of policy measures (scenarios) on the key variables considered, given the economic structure proposed by the DTC literature, but considering the public and private sectors. Thus, we consider that the production of final goods occurs in perfect competition in each sector and uses labor and a continuum of specific quality-adjusted intermediate goods. Intermediate goods, in turn, use prototypes (resulting from R &D activities) under monopolistic competition. Because of the close relationship between intermediate goods production and R &D, R &D can be encouraged either through direct subsidies or subsidies to intermediate goods production and can be discouraged by strong CIA constraints on R &D activity and intermediate goods production. Given the complementarity between factors of production and the substitutability between sectors, a new type of interrelationship between the above CIA constraints is introduced compared to the previous literature. Rational infinite-living dynastic households supply labor, maximize the utility obtained from the consumption of the final aggregate good, pay taxes, and obtain income from labor and investment in financial assets. In the proposed dynamic endogenous general equilibrium growth model, the aggregate final good is used in consumption and in investment.

Given that public sector workers are privileged,^3^ it was not without temptation to consider that they should also be sacrificed to free up resources to support the cuts borne by private sector workers. However, given that the public sector is a major employer, the prioritization of protecting all jobs, public and private,^4^ pointed to the need to avoid cuts in public employment or wages as a way to escape the deepening pandemic shock. The government understanding was therefore to consider the public sector wage bill as a natural countercyclical measure. Moreover, many public sector workers provide essential services in the response to the crisis; for example, according to World Bank data, about 25% are teachers and 15% are directly or indirectly linked to health services. Finally, any reduction in the public sector workforce during the crisis would hamper economic and social recovery; that is, short-term measures to reduce the workforce and/or wage cuts create long-term distortions.

As noted by Afonso and Pinho (2022), both empirical (Almus and Czarnitzki 2003; Gorg and Strobl 2007) and theoretical (Davidson and Segerstrom 1998; Zeng and Zhang 2007; Peretto 2003, 2007, 2011) studies point to the positive effects of direct and indirect R &D subsidies on economic growth, while the existing literature on the relationship between inflation and economic growth suggests otherwise (Benhabib and Spiegel 2009; Chu and Lai 2013; Chu et al. 2015). In general, there has been a positive impact of (direct and/or indirect) public intervention in R &D (Falk 2006), which has reduced the technological-knowledge gap between sectors and increased relative private sector wages. In line with the known data, the private sector is considered to have greater economic size and has recently been relatively more supported by policy measures, after having been relatively more penalized by the pandemic. The greater penalty during the pandemic justifies the differentiation in fiscal and monetary rules between sectors.

We study how such intervention, conducted by the government and the central bank, can ensure a significant increase (or not) in the degree of economic cohesion. If the relationships between both types of policies—fiscal (subsidies) and monetary (nominal interest rate)—and economic growth have been widely debated among economists, the same cannot be said of the relationships between, for example, both types of policies and the other key macroeconomic variables considered. Consequently, two important questions remain: Is the relationship between both types of policies and each of these variables—technological-knowledge gap, inter-sectoral wage inequality, and welfare—conclusive? Do the sign and intensity of each relationship depend on subsidies and inflation levels?

In addition to obtaining a negative relationship between inflation and the economic growth rate, the proposed model also records that private sector-oriented fiscal policy and/or an easing of CIA constraints for the private sector promote(s) a better balance across sectors in terms of technological knowledge, wage inequality, and social welfare. Despite losses in the short-to-medium run, the impact of monetary policy on economic growth, inflation, and welfare can be enhanced by introducing pecuniary transaction costs into consumption by introducing money into the utility function.

The paper is structured as follows. Section 2 describes the model. Section 3 determines the equilibrium conditions. Section 4 analyses the effects of governmental intervention through numerical analysis. Section 5 extends the model by considering money demand by households and firms. Finally, Sect. 6 offers some concluding remarks.

## Theoretical setup

### Overview

This Section describes the economic setup, emphasizing the interactions among economic agents.

The produced aggregate output (henceforth with the assignment of output), *Y*, can be used in consumption, *C*, investment in intermediate-goods productions, *X*, or in R &D expenditures, *R*, i.e., $Y=C+X+R$. The aggregate households decide between consumption and savings on income allocation. They inelastically supply labor, maximize utility of consumption and invest in the firm’s equity and money balances. The stylized model is composed of two representative sectors, public and private, each one composed of three productive subsectors: (i) a competitive final-goods/services (henceforth with the assignment of tasks) subsector; (ii) a monopolistic non-durable quality-adjusted intermediate-goods (henceforth with the assignment of intermediates) subsector; and (iii) a competitive R &D subsector. The tasks’ inputs are labor, which is perfectly mobile within each sector and immobile between sectors, and a continuum of specific quality-adjusted intermediates. As in Afonso and Forte (2021), for example, each intermediate, which is quality-adjusted, uses a design and output to be produced at a price chosen to maximize profits. In the R &D subsector, where designs are produced, each potential entrant devotes output to produce/invent a successful vertical design, which is then supplied to a new (monopolist) firm, which patent it; i.e., the R &D subsector allows to increase the quality of intermediates, and thus the technological knowledge, which improves the productivity of the sector in which is employed. Moreover, following Peretto (2007), Zeng and Zhang (2007), Atkeson and Burstein (2019), and Afonso and Forte (2021), among others, we consider that the government can influence the direction and progress of technological knowledge through its intervention in terms of tax/fiscal policy to assure a significant increase in the degree of economic cohesion. Finally, following Chu and Cozzi (2014), Gil and Iglésias (2019) and Afonso and Pinho (2022), among others, we incorporate money demand in the model via sector-specific cash-in-advance (CIA) constraint on R &D activities,^5^ and on the manufacturing of intermediate goods, whereas the monetary authority determines the money supply.

### Productive activity: technology and prices

Our model is an extension of Acemoglu and Zilibotti (2001), Afonso (2006), and Afonso and Forte (2021), retaining the same baseline assumptions. We define the output, *Y*,^6^ as a composite good of an infinite number of tasks thus resulting from:

1 $$\begin{aligned} Y = \int _{0}^{1}p_{n}Y_{n}\textrm{d}n \end{aligned}$$

where $p_{n}=\frac{P_{n}}{P_{Y}}$ is the real price of the task *n*,^7^$Y_{n}$ is the production of the task *n*, and $p_{n}Y_{n}$ is constant.^8^ The production of task *n* at time *t*, $Y_{n}(t)$, is then defined as:

2 $$\begin{aligned} Y_{n}&=\left[ \int _{0}^{J}(q^{k(j,t)}x_{n}(j,k,t))^{1-\alpha }\textrm{d}j\right] [(1-n)\cdot l\cdot L_{n}(t)]{}^{\alpha }\nonumber \\&\quad +\left[ \int _{J}^{1}(q^{k(j,t)}x_{n}(j,k,t))^{1-\alpha }\textrm{d}j\right] [n\cdot h\cdot H_{n}(t)]^{\alpha } \end{aligned}$$

where the integral terms represent the contributions of quality-adjusted intermediates to production. Sector *L*, the private, uses intermediates $j\in [0,J]$, while sector *H*, the public, requires other intermediates $j\in [J,1].$ The size of each quality upgrade due to successful R &D is denoted by *q*, an exogenously constant greater than 1. The rungs of the quality ladder are indexed by *k*, with higher *k*s denoting higher quality. At time 0, the highest quality good in each intermediate, *j*, has a quality index $k=0$. At *t* the highest quality produced by *j* has a quality index *k*(*j*, *t*), which is actually used due to profit maximizing limit pricing by the monopolist producers. The quantity $x_{n}(j,k,t)$ is the quantity of intermediate input *j* used together with the respective labor type, *L* or *H*, to produce $Y_{n}(t)$. The term $\left( 1-\alpha \right)$ is the aggregate intermediates input share and $\alpha \in \left( 0,1\right)$ is the labor share.

In each sector, the labor terms include the quantities of labor employed in the production of the *n*th task, $L_{n}$ or $H_{n}$, as well as two types of corrective sector accounting for productivity differentials. An absolute productivity advantage of the *H*-labor over the *L*-labor is accounted for by the parameter *h*, assuming $h>l\ge 1$; that is, *h* and *l* are positive exogenous variable representing the level of productivity, dependent on the average level of human capital that the respective workers have, which we consider higher in *H*; in the absence of estimates for this value in the literature, we consider the average value in empirical data for the US and European Union Member States over the last two decades for the long-run average human-capital level in the public and private sectors, noting that $h=1.10$. As in Afonso and Longras (2022), for example, a relative productivity advantage of either sector is captured by the terms *n* and $1-n$, which transform the index *n* into an ordering index meaning that the *H*-labor/sector is relatively more productive in producing tasks indexed by larger *n*’s, and vice-versa. As stated in Afonso and Longras (2022), since *n* $\in \left[ 0,1\right]$, there is an equilibrium threshold task, ${\overline{n}}(t)$, endogenously determined, where the switch from production in one sector to another becomes advantageous; moreover, the optimal choice of the producer sector is thus reflected in ${\overline{n}}(t)$, which results from profit maximization by perfectly competitive producers of the task and by intermediate monopolists, and by full-employment equilibrium in factor markets, given the labor supply in each sector and the current state of technological knowledge. In this sense, ${\overline{n}}(t)$ defines the sectoral structure and the comparative advantage of each sector in tasks since the production function combines complementarity between inputs with substitutability between the two sectors (e.g., Afonso and Longras 2022). That is, it consists of a weighted sum of two Cobb-Douglas production functions: if $Y_{n}$ is produced in the *L*-sector, factors are the *L*-labor and a specific set of quality-adjusted intermediate goods, while if $Y_{n}$ is produced in the *H*-sector factors are the *H*-labor and another specific set of quality-adjusted intermediate goods.

Due to zero profit equilibrium by producers of $n\in [0,1]$, the demand for the top-quality of *j* by the producer of *n* is

3 $$\begin{aligned} x_{n}(t)=\left[ \frac{p_{n}(t)(1-\alpha )}{p(j,k,t)}\right] ^{\frac{1}{\alpha }}q^{k(j,t)\left( \frac{1-\alpha }{\alpha }\right) }(1-n)\cdot l\cdot L_{n},\quad 0<j\le J, \end{aligned}$$

4 $$\begin{aligned} x_{n}(t)=\left[ \frac{p_{n}(t)(1-\alpha )}{p(j,k,t)}\right] ^{\frac{1}{\alpha }}q^{k(j,t)\left( \frac{1-\alpha }{\alpha }\right) }n\cdot h\cdot H_{n},\quad J<j\le 1, \end{aligned}$$

where: $p(j)=\frac{P(j)}{P_{Y}}$ is the real price of the intermediate *j*. A higher $p_{n}$ increases the marginal revenue product of the factors, encouraging firms to rent more intermediate. A higher $L_{n}$ or $H_{n}$ implies that more labor is used with intermediates, raising demand. Finally, a higher *p*(*j*) means lower demand, since the demand curve for intermediates is downward sloping. Plugging (3) and (4) into (2), i.e., considering the demand for each *j* by the producer of *n*, (2) can be written as

5 $$\begin{aligned} Y_{n}(t)=\left[ \frac{p_{n}(t)(1-\alpha )}{p(j,k,t)}\right] ^{\frac{1-\alpha }{\alpha }}\left[ (1-n)\cdot l\cdot L_{n}\cdot Q_{L}(t)+n\cdot h\cdot H_{n}\cdot Q_{H}(t)\right] , \end{aligned}$$

where $Q_{L}(t)\equiv \int _{0}^{J}q^{k(j,t)(\frac{1-\alpha }{\alpha })}\textrm{d}j$ and $Q_{H}(t)\equiv \int _{J}^{1}q^{k(j,t)(\frac{1-\alpha }{\alpha })}\textrm{d}j$ are aggregate quality indexes, measuring the technological knowledge in the sectoral-specific range of intermediates. Let us define $G\equiv \frac{Q_{H}}{Q_{L}}$, which accounts for the relative technological-knowledge level of *H*’s specific intermediates, giving a measure of the inter-sectoral technological-knowledge gap. An endogenous relevant result shown later is the level of *G*, which will allows us to analyze if a sector specific governmental intervention may improve its situation.

Following Afonso and Pinho (2022), since *Y* is the input in the production of $j\in [0,1]$ and tasks are produced in perfect competition, the real marginal cost of production of $j\in [0,1]$ is 1, regardless of the country. In terms of fiscal policy, we assume that the government can subsidize the production of *j* by paying an ad-valorem fraction, $z_{x}\in \left[ 0,1\right]$ (more specifically, $z_{x.L}$ in *L* and $z_{x,H}$ in *H*). Moreover, in terms of monetary policy, it is introduced a CIA constraint on the production of intermediates by assuming that firms use money, borrowed from households subject to the nominal interest rate $\iota (t)$, to pay for a fraction $\Omega _{x}\in \left[ 0,1\right]$ (more specifically, $\Omega _{x,L}$ in *L* and $\Omega _{x,H}$ in *H*), of the input; since firms cannot repay this amount to households until they earn revenue from production, households are effectively providing credit to these firms (e.g., Feenstra 1986). Hence, the after subsidy and CIA constraint marginal cost of producing *j* is $(1-z_{x}+\Omega _{x}\cdot \iota )$; i.e., the effective cost of the intermediate *j* is $(1-z_{x.L}+\Omega _{x,L}\cdot \iota )$ in *L* and $(1-z_{x.H}+\Omega _{x,H}\cdot \iota )$ in *H*.^9^ Following Romer (1990) and Afonso and Pinho (2022), *j* embodies a costly design (created in the R &D sector), which is recovered if profits at each date are positive for a certain time in future. This is assured by a patent law, which protects each leader firm’s monopoly, while at the same time, almost without costs, spreading acquired technological knowledge to other firms. The profit-maximization price of the monopolistic firms yields the constant over *t*, across *j* and for all *k* mark-up $p(k,j,t)=p=\frac{1-z_{x}+\Omega _{x}\cdot \iota }{1-\alpha }$, which, with $z_{x}-\Omega _{x}\cdot \iota <\alpha$, is in fact a mark-up over 1. Hence, as in Afonso and Forte (2021), for example, without any change in government intervention, this mark-up is stable over *t*, across *j* and for all *k*. This symmetry is thus dictated by the way in which each *j* enters in (2) and by the fact that all intermediate producers use the same input. Since the leader firm is the only one legally allowed to produce the top-quality, it will use pricing to wipe out lower quality sales. Depending on whether $q(1-\alpha )$ is greater or lesser than the real marginal cost 1, it will respectively use the monopoly pricing $p=\frac{1-z_{x}+\Omega _{x}\cdot \iota }{1-\alpha }$ or the limit pricing $p(k,j,t)=p=q\left( 1-z_{x}+\Omega _{x}\cdot \iota \right)$ to capture all the market. As in Grossman and Helpman (1991, Ch. 4), it is assumed that limit pricing strategy is used by all firms. Since the lowest price that the closest follower can charge without negative profits is $(1-z_{x}+\Omega _{x}\cdot \iota )$, the leader can capture all the market by selling at a price slightly below $q(1-z_{x}+\Omega _{x}\cdot \iota )$—more specifically, $q(1-z_{x,L}+\Omega _{x,L}\cdot \iota )$ in *L* and $q(1-z_{x,H}+\Omega _{x,H}\cdot \iota )$ in *H*—, because *q* represents the quality advantage over the closest follower.

In turn, the outcomes of R &D are designs, which improve the quality of intermediates and the quality indexes in (5) and thus drive economic growth in *L*-sector and *H*-sector, while creatively destroying the profits from previous advances (e.g., Aghion and Howitt 1992; Afonso and Pinho 2022), as the previous best quality loses that status. Let *I*(*j*, *k*, *t*) denote the instantaneous probability at *t*—a Poisson arrival rate—of successful innovation in the next higher quality $k(j,t)+1$ in *j*,

6 $$\begin{aligned} I_{L}(j,k,t)=y_{L}(j,k,t)\,\beta _{L}q^{k(j,t)}\,\varsigma _{L}^{-1}q^{k(j,t)\left( -\frac{1}{\alpha }\right) }\,L^{-\delta } \end{aligned}$$

7 $$\begin{aligned} I_{H}(j,k,t)=y_{H}(j,k,t)\,\beta _{H}q^{k(j,t)}\,\varsigma _{H}^{-1}q^{k(j,t)\left( -\frac{1}{\alpha }\right) }\,H^{-\delta } \end{aligned}$$

where: $y_{L}(k,j,t)$ and $y_{H}(k,j,t)$ are the flow of final-good resources devoted to R &D in *j* in sector *L* and in sector *H*; $\beta _{L}q^{k(j,t)}$ and $\beta _{H}q^{k(j,t)}$, $\beta _{L}>0$ and $\beta _{H}>0$, are the positive learning effect of accumulated public knowledge from past successful R &D in *j* in sector *L* and in sector *H*; $\varsigma _{L}^{-1}q^{k(j,t)\left( -\frac{1}{\alpha }\right) }$ and $\varsigma _{H}^{-1}q^{k(j,t)\left( -\frac{1}{\alpha }\right) }$, $\varsigma _{L}>0$ and $\varsigma _{H}>0$, are the adverse effect caused by the increasing complexity of quality improvements in *j* in sector *L* and in sector *H*;^10^$L^{-\delta }$ and $H^{-\delta }$, where $L=\int _{0}^{{\overline{n}}}L_{n}\textrm{d}n>H=\int _{{\overline{n}}}^{1}H_{n}{\rm d}n$, are the adverse effect of the market size in each sector, *L* and *H*, related to the difficulty of introducing new quality-adjusted intermediates and replacing old ones, and such difficulty is proportional to the size of the market due to coordination among agents, organizational and transportation costs, processing of ideas, information and marketing (e.g., Alesina and Spolaore 1997; Dinopoulos and Segerstrom 1999; Dinopoulos and Thompson 1999).

To sum up, R &D activities in each country result in innovative designs for intermediates’ production, which increase their quality. The designs are domestically patented and the leader in each *j*, which produces according to the latest patent, uses limit pricing to assure monopoly. The leading-edge patent’s value relies on the profit-yields accruing during each *t* to the monopolist, and on the monopoly-power duration. This one depends on the probability of an innovation, which creatively destroys the current leading-edge design in the lines of the Schumpeterian models (e.g., Aghion and Howitt 1992). Moreover, we will allow the government to subsidize R &D activities directly through an ad-valorem subsidy $z_{r}$, which can be sector-specific (i.e., $z_{r,L}$ in *L* and $z_{r,H}$ in *L*).

### Preferences and authorities

Following Bertinelli et al. (2013), Neto et al. (2019) and Afonso and Forte (2021), the representative infinitely-lived household maximizes the discounted intertemporal lifetime utility, which depends positively on its consumption and negatively on the labor level supplied, subject to the flow budget constraint and having perfect foresight concerning the technological-knowledge progress overtime; thus, at time $t=0$, the utility functions is $U=\int _{0}^{\infty }\left( \frac{C(t)^{1-\theta }-1}{1-\theta }-\frac{S^{1+\eta }}{1+\eta }\right) e^{-\rho t}\textrm{d}t$, whereby *U* is bounded away from infinity if the consumption of the output, $\left[ C(t)\right] _{t\ge 0}$, were stable over time, $\theta >0$ is the inverse of the inter-temporal elasticity of substitution, $\rho >0$ is the subjective discount rate, *S* represents *L* or *H*, $S=L,H$, and $\eta$ is the inverse of the Frisch elasticity (i.e., there is disutility from work).^11^ The representative infinitely-lived household collects income from investments in financial assets and in money balances (e.g., Chu and Cozzi 2014) and labor supply. The flow budget constraint is

8 $$\begin{aligned} {\dot{a}}(t)+{\dot{m}}(t)&=\left( 1-\tau _{a}\right) \cdot r(t)\cdot a(t)+\sum _{S=L,H}\left( 1-\tau _{w,S}\right) \cdot w_{S}(t)\cdot S-C(t)\nonumber \\&\quad +\tau (t)-\pi (t)\cdot m(t)+\iota (t)\cdot b(t) \end{aligned}$$

where: $a(t)=\sum _{S=L,H}a_{S}(t)$ is the household’s real financial assets/wealth holdings in the form of public debt owned by individuals and in the form of ownership of the firms that produce goods in monopolistic competition;^12^*r* is the real interest rate; $\tau _{a}$ is the ad-valorem tax on assets imposed by the government;^13^$w_{L}$ and $w_{H}$ are the wages paid in sector *L* and in sector *H*, and households inelastically supply labor, *L* or *H*; $\tau _{w,L}$ and $\tau _{w,H}$ are the governmental sector-specific ad-valorem taxes on wages,^14^ which, together with the governmental tax on financial assets $\tau _{a}$, may be used by the governments for a balanced government budget at each *t* in terms of domestic fiscal policies purposes (in particular, as a means of financing, at least partially, the costs of the above-mentioned subsidies); *m* is the households’ real money balances; $\tau$ denotes a lump-sum transfer/tax from the monetary authority—central bank; $\pi$ is the inflation rate, which determines the cost of holding money; *b* is the amount of money borrowed from households by intermediate firms to finance the manufacturing of intermediates and R &D investment, and which return is the nominal interest rate $\iota$. Thus, the CIA constraints imply that $b<m$. From standard dynamic optimization, we derive, respectively, a no-arbitrage condition between real money balances and real financial assets (this amounts to the well-known Fisher equation), the optimal path of consumption (the households’ Euler equation), and the optimal labor supply:

9 $$\begin{aligned} \iota (t)=\left( 1-\tau _{a}\right) r(t)+\pi (t), \end{aligned}$$

10 $$\begin{aligned} {\dot{C}}(t)=\frac{1}{\theta }\cdot \left[ \left( 1-\tau _{a}\right) \cdot r(t)-\rho \right] \cdot C(t), \end{aligned}$$

11 $$\begin{aligned} \frac{w_{H}}{w_{L}}=\left[ \frac{\left( 1-\tau _{w,H}\right) }{\left( 1-\tau _{w,L}\right) }\frac{H}{L}\right] ^{\eta } \end{aligned}$$

where $\frac{{\dot{C}}}{C}$ is the growth rate of *C*, and the transversality conditions are $\lim _{t\rightarrow +\infty }e^{-\rho t}\cdot C(t)^{-\theta }\cdot a(t)=0$ and $\lim _{t\rightarrow +\infty }e^{-\rho t}\cdot C(t)^{-\theta }\cdot m(t)=0$.^15^

As in Afonso and Pinho (2022), in addition to firms and individuals, the economy can also be influenced by the government policies and by the monetary authority or central bank. Hence, to finalize the characterization of the economy, a description of both the government’s budget and the monetary authority is now in order. As already stated, the government may intervene by imposing taxes on wages and/or on financial assets and by subsidizing the production of intermediate goods and/or R &D activities. We consider a balanced government budget, at each *t*, such that the budget surplus is 0:^16^

12 $$\begin{aligned} BuS=0\Rightarrow \tau _{a}\cdot r\cdot a+\sum _{S=L,H}\tau _{w,S}\cdot w_{S}\cdot S=\sum _{S=L,H}z_{x,S}\cdot X_{S}+\sum _{S=L,H}z_{r,S}\cdot R_{S}. \end{aligned}$$

Terms of the left-hand side of (12) represent the governments’ tax revenue from assets income and from labor income, while terms on right-hand side represent the governments expenditure on subsidies for intermediates and for R &D, assuming that there is no current public deficit. We will be particularly interested in the effects of higher levels of subsidies in *L*, regarding an eventual convergence towards the wage levels paid for *H*. Such an effect would become an argument in favor of different fiscal rules among sectors. As the paper’s main objectives are related to the aforementioned questions and taking into account the complexity of the model, the structure of public finances, described by (12), is simplified. In fact, we just consider some kind of public expenses, direct and indirect subsidies towards R &D,^17^ and some kind of taxes. It is sufficient to analyze the balance between economic cohesion and economic growth rate within the country.

In terms of the monetary authority we consider that it adopts an inflation-targeting framework. Its monetary policy instrument is the nominal interest rate, $\iota$, which affects the macroeconomic variables by operating through the CIA constraints (e.g., Chu and Cozzi 2014; Bernanke and Mishkin 1997; Afonso and Pinho 2022). Hence, with a change in the nominal interest rate, the CIA constraints generate forces that transmit different inflation costs, which distort the incentives and economic resources in the different sectors. We follow the literature and assume that the monetary authority exogenously chooses the nominal interest rate,^18^ so that $\iota (t)=\iota$. Thus, the inflation rate, $\pi (t)$, which corresponds to the growth rate of the nominal price of the output, $\pi (t)\equiv \frac{\dot{P_{Y}}(t)}{P_{Y}(t)}$, is endogenously determined according to the Fisher equation (9), for each *r*(*t*): $\pi (t)=\iota -\left( 1-\tau _{a}\right) r(t)$. Hence, in line with the empirical observation, which indicates that the inflation rate is endogenous and is indirectly controlled through the monetary policy instrument that is the nominal interest rate (e.g., Bernanke and Mishkin 1997), the Fisher equation reveals that the inflation rate is endogenous in the sense that relies on the real economic macroeconomic conditions reflected in the endogenous real interest rate. However, it is regulated by the exogenous choice of the nominal interest rate by the monetary authority. Denoting the nominal money supply by *M*(*t*), the real money supply/balances is $m(t)=\frac{M(t)}{P_{Y}(t)}$ and, in terms of growth rates, it results, respectively, that $\mu (t)\equiv \frac{{\dot{M}}(t)}{M(t)}$ and $\frac{{\dot{m}}(t)}{m(t)}=\mu (t)-\pi (t)$. Hence, knowing the value of $\pi (t)$ from the Fisher equation, the growth rate of the nominal money supply will be endogenously determined: $\mu (t)=\frac{{\dot{m}}(t)}{m(t)}+\pi (t)=\frac{{\dot{m}}(t)}{m(t)}+\iota -\left( 1-\tau _{a}\right) r(t)$.^19^ That is, the monetary authority will endogenously adjust the money growth rate to whatever level is needed for the interest rate $\iota$ to prevail. As usual in the literature, we consider that, to balance its budget, the monetary authority returns the seigniorage revenues to households as a lump-sum transfer, i.e., $\tau (t)=\frac{{\dot{M}}(t)}{P_{Y}(t)}=\frac{\dot{\left( m(t)\cdot P_{Y}(t)\right) }}{P_{Y}(t)}=\frac{{\dot{m}}(t)\cdot P_{Y}(t)+\dot{P_{Y}}(t)\cdot m(t)}{P_{Y}(t)}={\dot{m}}(t)+\pi (t)\cdot m(t)$.

As shown later on, the steady-state equilibrium relationships show that there is a relationship between the inflation rate, $\pi$, and the nominal interest rate, $\iota$, implying that we can extend all the comparative-statics results pertaining to shifts in $\iota$ also to shifts in the steady-state inflation rate, $\pi ^{*}$. Therefore, one could consider the inflation rate or even the growth rate of money supply as the policy variable directly controlled by the monetary authority. However, the consideration of the nominal interest rate as the policy instrument simplifies the analytical derivation of the steady-state equilibrium of the model without changing the comparative-statics results.

## Equilibrium

We proceed by analyzing the dynamic general equilibrium resulting from optimal decentralized behavior, which is described by the aggregate quality indexes’ equilibrium that drive economic growth. The interaction effects between *L*-sector and *H*-sector play thus a crucial role in the dynamic general equilibrium.

### Equilibrium for given technological knowledge

The competitive advantage of either sector on the production of the *n*th task relies on the relative productivity related to the average level of human capital incorporated in the two types of workers, $\frac{h}{l}$, and on the price of the sector-specific labor, as well as on the relative productivity and prices of the intermediates due to complementarity in production (e.g., Afonso and Longras 2022). The prices of labor depend on the quantities, *L* and *H*. In relative terms, the productivity-adjusted quantity of *H* in production is $\left( \frac{h\cdot H}{l\cdot L}\right)$. The productivity and prices of intermediates depend on complementarity with either labor, on the technological knowledge in the sector-specific range of intermediate goods and on the mark-up. These determinants are summed up in $Q_{L}$ and $Q_{H}$ in (5). The endogenous threshold final good ${\overline{n}}(t)$ follows from equilibrium in the inputs markets and relies on the determinants of the competitive advantage in tasks—see Appendix A:

13 $$\begin{aligned} {\overline{n}}(t)=\left\{ 1+\left[ G(t)\frac{h\, H}{{{l} }\,L}\right] ^{\frac{1}{2}}\right\} ^{-1}, \end{aligned}$$

In other words, since the production function (2) combines complementarity between inputs with substitutability between the two sectors, the optimal choice of technology is reflected in the equilibrium threshold task, ${\overline{n}}(t)$, which results from profit maximization (by perfectly competitive task producers and by intermediate monopolists) and full-employment equilibrium in factor markets, given the supply of labor and the current state of technological knowledge represented by *G* (e.g., Afonso and Forte 2021). Thus, the *H*-sector produces tasks $n>{\overline{n}}$ and the *L*-sector produces tasks $n\le {\overline{n}}$. The threshold can be related to prices noting that it is indifferent to produce it in *H* or *L*. This yields the ratio of index prices of tasks produced in each sector,

14 $$\begin{aligned} \frac{p_{H}(t)}{p_{L}(t)}=\left( \frac{{\overline{n}}(t)}{1-{\overline{n}}(t)}\right) ^{\alpha }. \end{aligned}$$

Moreover, taking into consideration that the output (1) is obtained by integration over final goods, and (13) and (14), the price-indexes of *L* and *H* tasks are, respectively,

15 $$\begin{aligned} p_{L}&=p_{n}\cdot \left( 1-n\right) ^{\alpha }=\exp \left( -\alpha \right) \cdot {\bar{n}}^{-\alpha }=\exp \left( -\alpha \right) \cdot \left\{ 1+\left[ G\frac{h\cdot H}{l\cdot L}\right] ^{\frac{1}{2}}\right\} ^{\alpha }\nonumber \\ p_{H}&=p_{n}\cdot n^{\alpha }=\exp \left( -\alpha \right) \cdot \left( 1-{\bar{n}}\right) ^{-\alpha }\nonumber \\&=\exp \left( -\alpha \right) \cdot \left\{ 1+\left[ G\frac{h\cdot H}{l\cdot L}\right] ^{-\frac{1}{2}}\right\} ^{\alpha } \end{aligned}$$

Equation (13) shows that a higher technological-knowledge gap, *G*, a larger relative supply of *H*-labor, $\frac{H}{L}$, and/or a higher productivity related to the average level of human capital incorporated in the two types of workers, $\frac{h}{l}$, results in a higher fraction of tasks produced in the *H*-sector, thus in a small ${\overline{n}}$; i.e., comparative advantage in more final goods. By (14), small implies a low relative price of final goods produced by *H*. In this case, the demand for *H* specific intermediate goods is relatively low, which discourages R &D activities to improve their quality. In particular, the labor structure affects the direction of R &D through the market-size and the price channels—in various papers by Acemoglu (e.g., 2002), the market-size channel dominates, which in Afonso (2006) and Afonso and Forte (2021) is removed and, consequently, becomes absent and therefore the price channel becomes the driver of the technological-knowledge bias.

The equilibrium aggregate resources devoted to intermediate-goods production, *X*, and the equilibrium aggregate output, *Y*, i.e., the composite final good in (1), are expressible as a function of the currently given aggregate quality indexes,^20^

16 $$\begin{aligned} X&=\exp (-1)\left[ \frac{\left( 1-\alpha \right) }{q\left( 1-z_{x}+\Omega _{x}\cdot i\right) }\right] ^{\frac{1}{\alpha }}\left[ \left( l\cdot L\cdot Q_{L}\right) ^{\frac{1}{2}}+\left( h\cdot H\cdot Q_{H}\right) ^{\frac{1}{2}}\right] ^{2}\quad \textrm{and}\nonumber \\ Y&=X\left[ \frac{\left( 1-\alpha \right) }{q\left( 1-z_{x}+\Omega _{x}\cdot i\right) }\right] ^{-1}, \end{aligned}$$

considering the simplifying case with $z_{x;L}=z_{x;H}$ and $\Omega _{x;L}=\Omega _{x;H}$. From the labor-demand perspective, the price paid for a unit of *s*-type labor, $w_{s}$, is equal to its marginal product and full-employment in the labor market, implicit in ${\overline{n}}$, yields the following equilibrium inter-sector wage inequality: $\frac{w_{H}(t)}{w_{L}(t)}=\left( G(t)\frac{h}{l}\frac{L}{H}\right) ^{\frac{1}{2}}$, which, combined with the labor-supply perspective in (11), gives rise to the following expression for inter-sector wage inequality—the sector-public premium—in the economy

17 $$\begin{aligned} W(t)=\frac{w_{H}(t)}{w_{L}(t)}=\left[ \left( G(t)\cdot \frac{h}{l}\right) ^{\eta }\cdot \frac{1-\tau _{w,L}}{1-\tau _{w,H}}\right] ^{\frac{1}{2\cdot \eta +1}}\text {.} \end{aligned}$$

### Equilibrium R &D

Given the functional forms (6) and (7) of the probabilities of success in R &D in each sector, free-entry equilibrium is defined by the equality between expected revenue and resources spent:

18 $$\begin{aligned} I_{S}(j,k,t)\cdot V_{S}(j,k,t)=(1-z_{r,S}+\Omega _{r,S}\cdot \iota )\cdot y_{S}(j,k,t),\quad \text {where:}\;{S=L,H} \end{aligned}$$

i.e., the present value of all the profit flows that a single innovator will receive for the discovery of a new quality intermediate good during the time s/he enjoys the detection (and consequent exploitation) of a patent in country *S*, $V_{S}$, times the probability of a new successful discovery, $I_{S}(k,j,t)$, must be equal to the effective R &D expenditures, $(1-z_{r,S}+\Omega _{r,S})\cdot y_{S}(k,j,t)$. As in Afonso and Forte (2021), for example, the effective R &D expenditures in sector *S* include: (i) a governmental subsidy to R &D, $z_{r,S}\cdot y_{S}$, where $z_{r,S}\in \left[ 0,1\right]$ is the ad-valorem subsidy thus reducing the R &D costs; and (ii) a financial component $\Omega _{r,S}\cdot \iota \cdot y_{S}$ that requires a share $\Omega _{r,S}\in \left[ 0,1\right]$ of money from households thus increasing the R &D costs.^21^

Hence, $V_{S}$ is the expected current value of the flow of profits to the monopolist producer of *j* or, in other words, the market value of the patent in sector *S*. The expected flow of profits depends on the amount at each *t*, $\Pi _{S}$, on the interest rate, and on the expected duration of the flow, which is the expected duration of technological-knowledge leadership. Such duration, in turn, depends on the probability of a successful innovation in *S*. The expression for $V_{S}$, $S=L,H$, is $V_{S}(j,t)=\int _{t}^{\infty }\Pi _{S}(j,t)\exp \left[ -\int _{t}^{s}\left( r(\nu )+I_{S}(j,\nu )\right) \textrm{d}\nu \right] \textrm{d}s$, which can be differentiated using Leibniz’s rule, resulting the following dynamic arbitrage equation:

19 $$\begin{aligned} r(\nu )+I_{S}(j,\nu )=\frac{\overset{.}{V_{S}}(j,t)}{V_{S}(j,t)}+\frac{\Pi _{S}(j,t)}{V_{S}(j,t)}-\overset{.}{k}(j,t)\left( \frac{1-\alpha }{\alpha }\right) \ln q. \end{aligned}$$

The equilibrium (18) can be translated into the equilibrium path of the technological-knowledge; in the case of sector *L* results:

20 $$\begin{aligned} \frac{{\dot{Q}}_{L}}{Q_{L}}&=\left\{ \frac{\beta _{L}}{\varsigma _{L}}\cdot \left( \frac{1-z_{x,L}+\Omega _{x,L}\cdot \iota }{1-z_{r,L}+\Omega _{r,L}\cdot \iota }\right) \left( \frac{q-1}{q}\right) \cdot l\cdot L^{1-\delta }\left[ \frac{p_{L}\left( 1-\alpha \right) }{1-z_{x,L}+\Omega _{x,L}\cdot \iota }\right] ^{\frac{1}{\alpha }}-r\right\} \nonumber \\&\quad \times \left( q^{\frac{1-\alpha }{\alpha }}-1\right) \end{aligned}$$

In (20), the term in large brackets is the equilibrium sector-specific probability of successful R &D, $I_{L}$, given *r* and $p_{L}$, which turns out to be independent of *j* and *k*, due to the removal of scale of technological-knowledge effects: the positive influence of the quality rung on profits and on the learning effect in (6) and (7) is exactly offset by its influence on the complexity cost also in (6) and (7). Additional scale/market effects could arise through market size, as has been intensely discussed in the R &D endogenous growth literature since Jones’ (1995) critique. Due to the technological complementarity in the production function (2), the size of the market for *L*-specific intermediate goods in our model is the level of labor *L*. Then, the scale effect is apparent in the size of the profits. Since we also aim at understanding the effects other than market size, the removal of scale is in order. The adverse effect of market size due to the scale-proportional difficulty of introducing new quality intermediate goods in (6) and (7) is designed to offset the scale effect on profits. With $\delta =1$, the offsetting is such that the influence of market size is null. Finally, it is clear that the total level of resources spent in R &D is

21 $$\begin{aligned} R=\int _{0}^{1}y(j)\cdot \textrm{d}j=\frac{\zeta }{\beta }\sum _{S=L,H}Q_{S}\cdot S^{\delta }\cdot I_{S} \end{aligned}$$

### Transitional dynamics and steady state

The stability properties of the transitional dynamics towards the steady state are block recursive, in the sense that we can first determine the stability of *G* and then recursively characterize the behavior of all the other variables.^22^ Bearing in mind that *r* is always unique in the economy and using $\frac{{\dot{Q}}_{H}}{Q_{H}}$ and $\frac{{\dot{Q}}_{L}}{Q_{L}}$ in (20) and (7) we can get the differential equation needed to obtain the path of the technological-knowledge gap between sectors, *G*, and then the behavior of other variables can be characterized:

22 $$\begin{aligned} \frac{{\dot{G}}}{G}&=\left( \frac{q-1}{q}\right) \left( q^{\frac{1-\alpha }{\alpha }}-1\right) \nonumber \\&\quad \cdot \left\{ \frac{\beta _{H}}{\varsigma _{H}}\cdot \left( \frac{1-z_{x,H}+\Omega _{x,H}\cdot \iota }{1-z_{r,H}+\Omega _{r,H}\cdot \iota }\right) \cdot h\cdot H^{1-\delta }\left[ \frac{p_{H}\left( 1-\alpha \right) }{1-z_{x,H}+\Omega _{x,H}\cdot \iota }\right] ^{\frac{1}{\alpha }}\right. \nonumber \\&\quad \left. -\frac{\beta _{L}}{\varsigma _{L}}\cdot \left( \frac{1-z_{x,L}+\Omega _{x,L}\cdot \iota }{1-z_{r,L}+\Omega _{r,L}\cdot \iota }\right) \cdot l\cdot L^{1-\delta }\left[ \frac{p_{L}\left( 1-\alpha \right) }{1-z_{x,L}+\Omega _{x,L}\cdot \iota }\right] ^{\frac{1}{\alpha }}\right\} . \end{aligned}$$

From (22) it is possible to explain on how governmental and monetary interventions affect the equilibrium. Take, for instance, an increase in subsidies in *L*. It re-directs R &D towards designs that improve relatively more its technological knowledge, which increases the relative productivity of its intermediate goods. The technological-knowledge gap between *L* and *H* falls, but this intervention creates/increases public deficit in *L*—see (12). The aim is to illustrate the effects of the governmental and monetary intervention on sector-specific technological knowledge. Using such results, we analyze how such intervention, when lead by the less developed sector, *L*, could improve the degree of economic cohesion within the country, without producing severe negative effects. In this context, we tackle whether different fiscal and monetary discipline rules may be needed to offset divergences in development between sectors.

The aggregate final good, *Y*, is used for consumption, *C*, and savings. Savings, in turn, are used in production of intermediate goods, *X*, and R &D activity, *R*.^23^ Since both sectors have access to the state-of-the-art intermediate goods and they have the same technology of production of tasks, in steady state they have differences in the levels but not in the growth rates. As in Afonso and Longras (2022), the common and stable steady-state growth rate is equal to the technological-knowledge progress, because *Y*, *X*, *R* and *C* are all constant multiples of $Q_{H}$ and $Q_{L}$. Through the Euler equation (10), the steady-state interest rate, $r^{*}(=r_{H}^{*}=r_{L}^{*})$, is also unique in the economy. The common and stable steady-state growth rate, designed by $g^{*}(=g_{H}^{*}=g_{L}^{*})$ is thus:

23 $$\begin{aligned} g^{*}&=\left( \frac{{\dot{Q}}_{S}}{Q_{S}}\right) ^{*}=\left( \frac{{\dot{Y}}}{Y}\right) ^{*}=\left( \frac{{\dot{X}}}{X}\right) ^{*}=\left( \frac{{\dot{R}}}{R}\right) ^{*}=\left( \frac{{\dot{C}}}{C}\right) ^{*}=\left( \frac{{\dot{c}}}{c}\right) ^{*}\nonumber \\&=\frac{\left( 1-\tau _{a}\right) \cdot r^{*}-\rho }{\theta }, \end{aligned}$$

implying constant steady-state levels of $G^{*}$. Thus, $r^{*}$ is obtained by setting the growth rate of consumption in (10) equal to the growth rate of technological knowledge in (20), and using the equilibrium levels of $p_{H}$ and $p_{L}$. Clearly, R &D drives steady-state endogenous growth. If $G(0)=\frac{Q_{H}(0)}{Q_{L}(0)}$ is such that $\frac{{\dot{G}}(0)}{G(0)}>0$, ${\overline{n}}$ will decrease overtime, which implies that$\frac{\dot{p_{H}}}{p_{H}}<0$ and $\frac{\dot{p_{L}}}{p_{L}}>0$. In turn, a lower $p_{H}$ and a higher $p_{L}$ over time induce lower incentives to perform R &D directed to the *H*-sector in relation to the *L*-sector, which implies a lower $\frac{{\dot{G}}}{G}$. These dynamics continue until $\frac{{\dot{G}}}{G}=0$. If *G*(0) is such that $\frac{{\dot{G}}(0)}{G(0)}<0$, the opposite dynamics will occur.

From (20) and (14), $Q_{L}$ and $Q_{H}$ rise at the same rate when:

24 $$\begin{aligned} {\overline{n}}^{*}&=\left[ 1+\frac{\beta _{H}}{\beta _{L}}\cdot \frac{\varsigma _{L}}{\varsigma _{H}}\cdot \left( \frac{1-z_{r,L}+\Omega _{r,L}\cdot \iota }{1-z_{r,H}+\Omega _{r,H}\cdot \iota }\right) \cdot \left( \frac{1-z_{x,L}+\Omega _{x,L}\cdot \iota }{1-z_{x,H}+\Omega _{x,H}\cdot \iota }\right) ^{\frac{1-\alpha }{\alpha }}\cdot \frac{h}{l}\right. \nonumber \\&\quad \cdot \left. \left( \frac{H}{L}\right) ^{1-\delta }\right] ^{-1}. \end{aligned}$$

Then, bearing in mind (13) and (24), the steady-state inter-sector technological-knowledge gap is:

25 $$\begin{aligned} G^{*}&=\left[ \frac{\beta _{H}}{\beta _{L}}\cdot \frac{\varsigma _{L}}{\varsigma _{H}}\cdot \left( \frac{1-z_{r,L}+\Omega _{r,L}\cdot \iota }{1-z_{r,H}+\Omega _{r,H}\cdot \iota }\right) \cdot \left( \frac{1-z_{x,L}+\Omega _{x,L}\cdot \iota }{1-z_{x,H}+\Omega _{x,H}\cdot \iota }\right) ^{\frac{1-\alpha }{\alpha }}\right] ^{2}\nonumber \\&\quad \cdot \frac{h}{l}\cdot \left( \frac{H}{L}\right) ^{1-2\cdot \delta }. \end{aligned}$$

In turn, from (17) and (25), the steady-state inter-sector wage inequality—sector-public premium—is:

26 $$\begin{aligned} W^{*}=\left( \frac{w_{H}}{w_{L}}\right) ^{*}=\left( G^{*}\cdot \frac{h}{l}\right) ^{\frac{\eta }{2\cdot \eta +1}}\left[ \left( G^{*}\cdot \frac{h}{l}\right) ^{\eta }\cdot \frac{1-\tau _{w,L}}{1-\tau _{w,H}}\right] ^{\frac{1}{2\cdot \eta +1}}. \end{aligned}$$

Finally, choosing the case of the *L*-sector and bearing in mind (20) and (23), we first determine the stable and unique steady-state interest rate and then we insert that expression in (23) to determine the steady-state economic growth rate:

27 $$\begin{aligned} g^{*}=\frac{\left( 1-\tau _{a}\right) \cdot \frac{\beta _{L}}{\varsigma _{L}}\cdot \left( \frac{1-z_{x,L}+\Omega _{x,L}\cdot \iota }{1-z_{r,L}+\Omega _{r,L}\cdot \iota }\right) \left( \frac{q-1}{q}\right) \cdot l\cdot L^{1-\delta }\left[ \frac{p_{L}^{*}\left( 1-\alpha \right) }{1-z_{x,L}+\Omega _{x,L}\cdot \iota }\right] ^{\frac{1}{\alpha }}-\rho }{\left( q^{\frac{1-\alpha }{\alpha }}-1\right) ^{-1}+\theta }, \end{aligned}$$

where $p_{L}^{*}$ can be determined by the expressions (15) and (24), $p_{L}^{*}=\exp (-\alpha )\cdot {\overline{n}}^{*-\alpha }$. In the determination process for $g^{*}$, $r^{*}$ was obtained first; thus, by (9) the steady-state inflation rate is:

28 $$\begin{aligned} \pi ^{*}=\iota -\left( 1-\tau _{a}\right) r^{*}\Leftrightarrow \pi ^{*}=\iota -\rho -\theta g^{*}. \end{aligned}$$

We can also compute the welfare measure, $Z_{S}$, obtained from the lifetime utility function:

29 $$\begin{aligned} Z_{S}^{*}=\frac{1}{1-\theta }\left\{ \frac{C(0)^{1-\theta }}{\left[ \rho -\left( 1-\theta \right) g^{*}\right] }-\frac{1}{\rho }\right\} -\frac{S^{1+\eta }}{\left( 1+\eta \right) \rho }. \end{aligned}$$

Bearing in mind expressions (24), (25), (26), (27), (28) and (29), standard comparative-statics techniques yield the following Table 1 concerning changes in the structural parameters, the exogenous fiscal and monetary policy variable, as well as exogenous variables. The results of Table 1 are explained in Proposition 1.

**Table 1:** Effect of a permanent and unanticipated increase of each structural parameter, fiscal and monetary parameter, and exogenous variable on the steady-state value of the main variables. The effects of $\iota$ are based on the starting situation in which $z_{r,L}=z_{r,H}$, $z_{x,L}=z_{x,H}$, $\Omega _{r,L}=\Omega _{r,H}$ and $\Omega _{x,L}=\Omega _{x,H}$

	Structural parameters, fiscal and monetary parameters, and exogenous variables								
	$\alpha$, $\tau _{a}$, $\iota$	*h*, $\beta _{H}$, $\delta$, $z_{r,H}$, $z_{x,H}$	*l*, $\beta _{L}$, $z_{r,L}$, $z_{x,L}$	$\theta$, $\rho$	$\eta$	$\varsigma _{L}$, $\Omega _{r,L}$, $\Omega _{x,L}$	$\varsigma _{H}$, $\Omega _{r,H}$, $\Omega _{x,H}$	*L*	*H*
${\frac{\partial {\overline{n}}^{*}}{\partial (\cdot )}}$	0	−	+	0	0	−	+	+	−
${\frac{\partial G^{*}}{\partial (\cdot )}}$	0	+	−	0	0	+	−	+	−
${\frac{\partial W^{*}}{\partial (\cdot )}}$	0	+	−	0	+	+	−	+	−
${\frac{\partial g^{*}}{\partial (\cdot )}}$	−	+	+	−	0	−	−	+	+
${\frac{\partial \pi ^{*}}{\partial (\cdot )}}$	+	−	−	−	0	+	+	−	−
${{\frac{{\partial Z_{L}^{*}}}{{\partial (\cdot )}}}}$	−	+	+	−	+	−	−	−	+
$\frac{\partial Z_{H}^{*}}{\partial (\cdot )}$	−	+	+	−	+	−	−	+	−

#### Proposition 1

*In terms of structural parameters, an increase in*: (A) *the labor share*, $\alpha$, *penalizes the economic growth rate*, $g^{*}$, *and the welfare*, $Z_{S}^{*}$, *and generates inflation*, $\pi ^{*}$; (B) (*a decrease in*) *h* *or* $\beta _{H}$ (*l* *or* $\beta _{L}$) *increases the sector-public premium*, $W^{*}$, *decreases the threshold final good*, ${\overline{n}}^{*}$, *and an increase in any of these parameters increases the economic growth rate*, $g^{*}$, *and the welfare*, $Z_{S}^{*}$, *and decreases the inflation rate*, $\pi ^{*}$; (C) (*a decrease in*) $\varsigma _{H}$ ($\varsigma _{L}$) *decreases the sector-public premium*, $W^{*}$, *increases the threshold final good*, ${\overline{n}}^{*}$, *and an increase in any of these parameters decreases the economic growth rate*, $g^{*}$, *and the welfare*, $Z_{S}^{*}$, *and increases the inflation rate*, $\pi ^{*}$; (D) *the removal scale-effects parameter*, $\delta$, *produces effects similar to those arising from an increase in* *h* *or* $\beta _{H}$; (E) *the inverse of the inter-temporal elasticity of substitution*, $\theta$, *or in the subjective discount rate*, $\rho$, *affect negatively the economic growth rate*, $g^{*}$; (F) *the inverse of the Frisch elasticity elasticity*, $\eta$, *favors the public-sector premium*, $W^{*}$, *and the welfare*, $Z_{S}^{*}$.

*In terms of policy effects, an increase in*: (A) (*a decrease in*) $\Omega _{r,H}$ *or* $\Omega _{x,H}$ ($\Omega _{r,L}$ *or* $\Omega _{x,L}$) *decreases the sector-public premium*, $W^{*}$, *increases the threshold final good*, ${\overline{n}}^{*}$, *and an increase in any of these parameters decreases the economic growth rate*, $g^{*}$, *and the welfare*, $Z_{S}^{*}$, *and increases the inflation rate*, $\pi ^{*}$; (B) *the nominal interest rate*, $\iota$, *by the central bank or the ad-valorem tax on assets*, $\tau _{a}$, *affect negatively the economic growth rate*, $g^{*}$, *and* *the welfare*, $Z_{S}^{*}$, *and decreases the inflation rate*, $\pi ^{*}$; (C) (*a decrease in*) $z_{r,H}$ *or* $z_{x,H}$ ($z_{r,L}$, *or* $z_{x,L}$) *increases the sector-public premium*, $W^{*}$, *decreases the threshold final good*, ${\overline{n}}^{*}$, *and an increase in any of these parameters increases the economic growth rate*, $g^{*}$, *and the welfare*, $Z_{S}^{*}$, *and decreases the inflation rate*, $\pi ^{*}$.

*In terms of labor levels, an increase in the labor level* *H* (*L*) *decreases* (*increases*) *the sector-public premium*, $W^{*}$, *the threshold final good*, ${\overline{n}}^{*}$, *and the welfare*, $Z_{H}^{*}$ ($Z_{L}^{*}$), *and an increase in any of these parameters increases the economic growth rate*, $g^{*}$, *and decreases the inflation rate*, $\pi ^{*}$.

#### Proof

Check the partial derivatives of Eqs. (24), (25), (26), (27), (28) and (29) with respect to structural parameters, fiscal and monetary parameters, and exogenous variables of the model. $\square$

In addition to the content of Proposition 1, the following considerations are worth highlighting or reinforcing. The effects on the public-sector premium come from labor supply and what can affect it (e.g., the inverse of the Frisch elasticity elasticity), and from labor demand. This, in turn, is mainly motivated by the technological-knowledge gap, *G*, which, when it increases (decreases), biasing towards the *H*-sector (*L*-sector) favors its relative wage. An increase of the labor share makes R &D less productive, thus penalizing economic growth and welfare,^24^ and increasing the inflation rate. In turn, an increase in one corrective measure of labor productivity, *h* or *l*, or in one learning-by-past parameter, $\beta _{H}$ or $\beta _{L}$, makes R &D activity more productive, increasing the growth rate and welfare, and decreasing the inflation rate. The effects of an increase in the removal scale-effects parameter, $\delta$, are, as expected, similar to those of an increase in *h* or $\beta _{H}$ because, with $L>H$, a greater removal of scale effects is equivalent to a greater benefit for less abundant labor. Moreover, an increase in the inverse of the inter-temporal elasticity of substitution, $\theta$, or in the subjective discount rate, $\rho$, affect negatively the economic growth rate,^25^ welfare and inflation. According to the supply–demand law, an increase in the inverse of the Frisch elasticity elasticity favors the most scarce labor and, therefore, the public-sector premium, as well as the welfare. In terms of policy effects, an increase in the parameters associated with the monetary CIA constraints of a sector, $\Omega _{r,L}$, $\Omega _{x,L}$, $\Omega _{r,H}$, or $\Omega _{x,H}$, decreases economic growth and welfare, while generating inflation. The same effects on the economy—i.e., on growth, welfare and inflation—emerge following an increase in the nominal interest rate, $\iota$, by the central bank or the ad-valorem tax on assets, $\tau _{a}$, by the government since these changes—directly or indirectly—make R &D less profitable. For example, reflecting the negative relationship between $r^{*}$ and $\iota$, we find a negative relationship between the long-run economic growth rate, $g^{*}$, and $\iota$.^26^ In turn, a more generous fiscal policy due to an increase in $z_{r,L}$, $z_{x,L}$, $z_{r,H}$, or $z_{x,H}$, increases the economic growth and welfare, in parallel with the penalization of the inflation growth rate.

#### Proposition 2

*The long-run inflation rate*, $\pi ^{*}$, *is an increasing function of the exogenous monetary policy variable*, $\iota$.

#### Proof

It results directly from (28) since the partial derivative of $g^{*}$ with respect to $\iota$ is negative—see Appendix C. $\square$

As mentioned in the introduction and as is well known, the COVID-19 pandemic has paralyzed the world economy and represents an immense shock on the economy of the different countries (e.g., Adams-Prassl et al. 2020). More than one third of the world’s population has been placed in isolation, activities involving groups of people have been suspended and a significant number of workers have fallen ill. In particular, it was the private sector of each economy that lost most income due to firms’ production stoppage (Thompson 2020), with significant wage cuts—see the case of wage cuts motivated by lay-offs. The economic impacts of the crisis on the private and public sectors are, therefore, distinct and, as already stated, this distinction motivated this paper. As a result of this evidence, the shock induced by the pandemic has manifested in all sectors, but it has clearly manifested more in the private sector. Thus, in sectoral terms, we need to consider that, in relative terms, there have been losses in the private sector. Here, we consider that there were losses in (2), materialized in the decrease in labor productivity associated with the private sector such that *l* decreases—a negative labor productivity shock due to the COVID-19 pandemic. Moreover, another feature of our the shock is also reflected in R &D technology (6) and (7), and we assume that the learning-by-past parameter $\beta _{L}$ also suffered a sharp reduction. Consequently, in line with Table 1 (column 3 of results) and the reality of the shock, in the new steady state the number of final goods supplied by the private sector, the economic-growth rate and welfare decrease, in parallel with the increase in the inflation rate, the technological-knowledge bias towards the public sector and the public-sector premium.

To reverse this new situation the government should conduct a fiscal policy by subsidizing (direct or indirect) R &D in sector *L*, thus counterbalancing the effects of the shock (Table 1, column 3), and/or the central bank should conduct a monetary policy to ease CIA constraints in the *L*-sector. The long-run relationship between policy instruments and each real macroeconomic variables of interest is independent of the relative degree of the instruments on R &D activity vis-a-vis production of intermediate goods.

## Numerical analysis

### Data and calibration

Now, we solve numerically the transition dynamics to the steady state and the steady-state values of the variables of interest to illustrate the effect of the COVID-19 shock as well as the subsequent effects of policy intervention on the specific sector. Using these results, we analyze whether different fiscal and monetary rules may be necessary (or not), for example, to compensate for divergences between sectors and to restore the pre-shock situation. The transitional dynamics is solved through the fourth-order Runge–Kutta classical numerical method, which solves (22) with suitable precision. The time path of technological-knowledge gap is displayed, bearing in mind the baseline parameter values and labor endowments. This way, the transitional dynamics is displayed, bearing in mind the initial condition $G(0)=1$ (i.e., we assume that initially there is no technological-knowledge bias) and the set of baseline parameter values and labor endowments in Table 2, necessary to analyze the variables of interest.

We follow the traditional approach of the literature and established the usual values for $\theta$, $\rho$, and $\alpha$ (e.g., Jones and Williams 2000; Gil and Iglésias 2019; Afonso and Pinho 2022). Following the same procedure as Afonso (2008) and Afonso and Pinho (2022), as a starting point, the values of the parameters associated to the policy measures were considered equal to 0 in the baseline scenario; the idea is to stay within a ‘consensus benchmark’ and to leave further discussion for the policy intervention analysis and also because the same steady-state values for the variables of interest would be obtained considering $z_{r,L}=z_{r,H}$, $z_{x,L}=z_{x,H}$, $\Omega _{r,L}=\Omega _{r,H}$ and $\Omega _{x,L}=\Omega _{x,H}$. The inverse of the Frisch elasticity $\eta$ is in line with Chetty (2012) and Bertinelli et al. (2013), among others. The values for the remaining parameters and exogenous variables—*h*, *l*, $\beta _{H}$, $\beta _{L}$, $\varsigma _{H}$, $\varsigma _{L}$, $\delta$, $\iota$, *L*, *H*, and *C*(0)—were considered taking into account the values of the R &D sector parameters in the literature (Gil and Iglésias 2019; Afonso and Magalhães 2020), and the need to match the average values of the empirical data for the USA and for the European Union Member States over the last two decades as regards the long-run: (i) public-sector wage premium, $W_{\textrm{observed}}^{*}=1.11$, employment in the public and private sectors, $\left( \frac{H}{L}\right) _{\textrm{observed}}=0.25$, level of average human capital in the public and private sectors, $\left( \frac{h}{l}\right) _{\textrm{observed}}=1.1$ (Christofides and Michael 2013; Flannery and Turner 2018; Dolton et al. 2020; Michael and Christofides 2020, and available Eurostat data and OECD data); (ii) economic-growth rate, $g_{\textrm{observed}}^{*}=2.08{\%}$, inflation rate, $\pi _{\textrm{observed}}^{*}=2.88{\%}$ and real interest rate, $r_{\textrm{observed}}^{*}=5.12{\%}$ (Chu et al. 2017; Gil and Iglésias 2019, and available Eurostat data and OECD data). In particular, the value for *C*(0) was normalized to 1.

**Table 2:** Structural parameter, fiscal and monetary parameter, and exogenous variable—values used in the model

Parameter/variable	Description	Value	Parameter/variable	Description	Value
$\alpha$	Labor share	0.60	$\eta$	Inverse of the Frisch elasticity	1.00
*L*	Labor in private sector	1.00	$\tau _{a}$	Tax on financial assets	0.00
*H*	Labor in public sector	0.25	$\Omega _{x,L}$	CIA constraint on *L*-intermediate goods	0.00
*l*	*L*-labor productivity	1.00	$\Omega _{x,H}$	CIA constraint on *H*-intermediate goods	0.00
*h*	*H*-labor productivity	1.10	$\Omega _{r,L}$	CIA constraint on *L*-R &D	0.00
$q=\frac{1}{1-\alpha }$	Size of each quality upgrade	2.50	$\Omega _{r,H}$	CIA constraint on *H*-R &D	0.00
$\beta _{L}$	*L*-learning-by-past R &D	1.00	$z_{x,L}$	Subsidy to the *L*-intermediate goods	0.00
$\beta _{H}$	*H*-learning-by-past R &D	2.10	$z_{x,H}$	Subsidy to the *H*-intermediate goods	0.00
$\varsigma _{L}$	*L*-R &D complexity	1.10	$z_{r,L}$	Subsidy to the *L*-R &D	0.00
$\varsigma _{H}$	*H*-R &D complexity	3.10	$z_{r,H}$	Subsidy to the *H*-R &D	0.00
$\delta$	Adverse market-size effect	0.8	$\iota$	Nominal interest rate	0.08
$\rho$	Subjective discount rate	0.02	*C*(0)	Initial consumption	1.00
$\theta$	Inverse of inter-temporal elasticity of substitution	1.50	*G*(0)	Initial technological-knowledge gap	1.00

To better understand the theoretical model and its results, let us assume, in numerical terms, that the pandemic caused a drop of *l* to 0.9 and of $\beta _{L}$ also to 0.9, and we consider five alternative scenarios after the shock: (i) Scenario 1 (Sc1)—no public intervention occurs; (ii) Scenario 2 (Sc2)—an ad-valorem subsidy to the production of intermediate goods in sector *L*, $z_{x,L}=0.2$, is introduced; (iii) Scenario 3 (Sc3)—an ad-valorem subsidy to the production of intermediate goods and to R &D in sector *L*, $z_{x,L}=z_{r,L}=0.1$, are considered; (iv) Scenario 4 (Sc4)—the monetary authority acts and, on the one hand, in line with Brown and Petersen (2015) and Gil and Iglésias (2019), $\Omega _{x}>\Omega _{r}$, and, on the other hand, in line with Pagano et al. (1998) and Saunders and Steffen (2011), $\Omega _{L}>\Omega _{H}$,^27^ thus it is assumed that $\Omega _{r,L}=0.8>\Omega _{r,H}=0.7>\Omega _{x,L}=0.5>\Omega _{x,H}=0.4$, while the government proceeds according to Sc3 $z_{x,L}=z_{r,L}=0.1$; (v) Scenario 5 (Sc5)—illustrates the case where alternatively $\Omega _{r,L}=\Omega _{r,H}=0.7>\Omega _{x,L}=\Omega _{x,H}=0.4$ and $z_{x,L}=z_{r,L}=0.1$. In this latter scenario, the decrease of $\Omega _{r,L}$ and $\Omega _{x,L}$ compared to the previous scenario is equivalent to a decrease in the nominal interest rate, $\iota$, from Sc4 to Sc5. When considering different scenarios, two exclusively with fiscal policy (scenarios 2 and 3) and others in which we added monetary policy (scenarios 4 and 5), we also took into account the IMF procedure when, during the COVID-19 emergency period, it divided fiscal and monetary policies into several aspects for all countries.

### Short-medium-long-run results

**Fig. 1:**
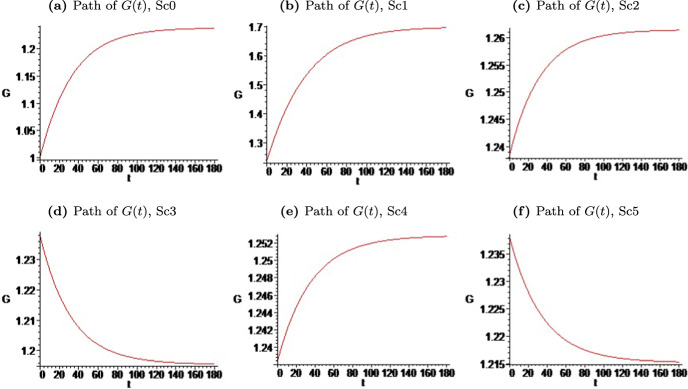
Transitional dynamics of *G*(*t*) in all scenarios

Using the fourth-order Runge–Kutta classical numerical method, in Fig. 1 we present the precise time path of the technological-knowledge gap between sectors or the relative productivity of the technological knowledge used together with *H*, measured by the path of *G*, bearing in mind the set of baseline parameter values and exogenous variables in Table 2. Once characterized the behavior of *G* during the transition dynamics to the steady state, then the behavior of other variables, namely the *H*-premium or public-sector premium in (17), could also be easily illustrated. By reason of complementarity between inputs in (2), changes in the *H*-premium are closely related to the technological-knowledge bias, as (17) clearly shows. Figure 1a shows the transition dynamics to the steady state (supposedly) existing in the pre-shock period, taking into account the values of the parameters and exogenous variables and considering one as initial value of *G*, $G(0)=1$, although the steady-state value achieved is independent of the initial value. Thus, as shown in Table 3, it is considered that, in the pre-shock period, the country was in the steady state, with about 63% of final goods to be supplied by the private sector, with a public sector wage premium of 11%, an economic growth rate of 2.08%, the inflation rate around 2.88%, and the social welfare levels given by $Z_{L}^{*}=17.76$ and $Z_{H}^{*}=33.18$ for the private and public sectors, respectively. That is, Scenario 0, Sc0, or pré-COVID-19 time in Table 3 corresponds to the steady-state that the economy was supposed reached when the shock caused by the pandemic emerged and the first column of Table 3 provides the values of the variables of interest.

As mentioned above, the pandemic caused an immediate drop in *l* and $\beta _{L}$, whose values have gone from 1 to 0.9 and, consequently, despite the maintenance of the stock variable $G_{{\text {Pre-COVID-19}}}^{*}=1.24$, the shock affected the remaining variables. In particular, at the time of the shock, from (13) and (17), we find ${\overline{n}}_{{\text {COVID-19}}}=0.62$ and $\left( \frac{w_{H}}{w_{L}}\right) _{{\text {COVID-19}}}=1.15$; i.e., due to the change in *l* and without new endogenous technological-knowledge progress and so without change in technological-knowledge bias, the threshold final good () and the *H*-premium increase instantly—see (13) and (17). Moreover, from (16), we calculated the values for $Y_{{\text {Pre-COVID-19}}}^{*}$ and $Y_{{\text {COVIT-19}}}$, considering, by simplification, that $Q_{L}^{*}=1$ and $Q_{H}^{*}=1.24$ since $G_{{\text {Pre-COVID-19}}}^{*}=G_{{\text {COVID-19}}}=1.24$, and taking into account, obviously, the impact of the shock; with this procedure resulted that $g_{{\text {COVID-19}}}=\ln \left( \frac{Y_{{\text {COVID-19}}}}{Y_{{\text {Pre-COVID-19}}}^{*}}\right) =-6.59{\%}$. Furthermore, from $\pi =\iota -\rho -\theta g_{C}$ and considering that $g_{{\text {COVID-19}}}=\left( \frac{{\dot{Y}}}{Y}\right) _{{\text {COVID-19}}}=\left( \frac{{\dot{C}}}{C}\right) _{{\text {COVID-19}}}$, we have $\pi _{{\text {COVID-19}}}=3.39{\%}$.

After the shock a number of scenarios can then occur, according to the policy measures taken, and Table 3 summarizes the steady-state results for the different cases. Scenario 1, Sc1, corresponds to considering that there is neither government intervention nor central bank intervention. Due to the decrease in *l* and $\beta _{L}$, the economic incentive to make technological-knowledge progress in the private sector decreases, either because it becomes relatively less profitable—the demand for intermediate goods in the private sector drops due to the fall of *l*, as can be seen in (3)—or because it becomes relatively more painful—as can be seen in (7). This heightens the technological-knowledge bias in favor of *H*-intermediate goods—see Fig. 1b. Such bias increases the supply of *H*-intermediate goods, thereby increasing the number of final goods produced with *H*-technology—i.e., decreasing ${\overline{n}}$; see (13)—and lowering their relative price—see (14). Thus, relative prices of final goods produced with *H*-technology drop continuously towards the constant steady-state levels. This path of relative prices implies that the technological-knowledge bias is increasing, from $G_{{\text {Pre-COVID-19}}}^{*}$, but at a decreasing rate until it reaches its new higher steady state, $G_{{\text {Sc1}}}^{*}=1.70$—see Fig. 1b and Table 3. As stated above, a decrease of *l* due to the negative labor productivity shock as a result of the COVID-19 pandemic causes an immediate increase in the *H*-premium, from $\left( \frac{w_{H}}{w_{L}}\right) _{{\text {Sc0}}}^{*}=1.11$ to $\left( \frac{w_{H}}{w_{L}}\right) _{{\text {COVID-19}}}=1.15$—see (17). In other words, the *H*-premium increases instantly due to the fall in the labor productivity of *L*. As the decrease in *l* (and in $\beta _{L}$) induces technological-knowledge bias, the immediate effect on the level of the *H*-premium is enhanced during the transition dynamics towards the steady state. That is, the stimulus to the demand for *H*, arising from the technological-knowledge bias, increases the *H*-premium. Once in steady state, with a constant technological-knowledge bias, the *H*-premium remains constant. The loss to the economy that represented the fall in *l* and $\beta _{L}$ resulted in a decrease in the technological-knowledge progress and, consequently, in the economic growth rate, $g_{{\text {Sc0}}}^{*}=2.08{\%}>g_{{\text {Sc1}}}^{*}=1.74{\%}>g_{{\text {COVID-19}}}=-6.59{\%}$, as well as in the increase in the inflation rate $\pi _{{\text {Sc0}}}^{*}=2.88{\%}<\pi _{{\text {Sc1}}}^{*}=3.39{\%}\simeq \pi _{{\text {COVIT-19}}}=3.39{\%}$ and in the degradation of the welfare. At this level, the degradation was more intense, as expected, in the private sector: $\ln \left( \frac{Z_{L,{\text {Sc1}}}=12.91}{Z_{L,{\text {Sc0}}}=17.76}\right) =-32{\%}<\ln \left( \frac{Z_{H,{\text {Sc1}}}=29.24}{Z_{H,{\text {Sc0}}}=33.18}\right) =-13{\%}$.

In the face of the shock, Sc2 and Sc3 consider that there is government intervention in favor of the private sector, which is captured by direct R &D subsidies, $z_{r,L}$, thus reducing the associated R &D costs, or by encouraging the production of the respective intermediate goods, $z_{x,L}$, thus indirectly stimulating investment in the private sector R &D. In particular, Fig. 1c, d show the paths of *G* until the new steady-state as a result of an exogenous increase of just $z_{x,L}=0.2$ in Sc2, and of both $z_{x,L}=0.1$ and $z_{r,H}=0.1$ in Sc3. These Subfigures reveal that *G* becomes more biased towards the *L*-sector in both cases ($G_{\textrm{Sc1}}^{*}=1.70>G_{\textrm{Sc2}}^{*}=1.26>G_{\textrm{Sc3}}^{*}=1.20$). Indeed, since a greater $z_{x,L}$ increases the size of profits that accrue to the producers of *L*-type intermediate goods, and a greater $z_{r,L}$ decreases the cost of *L*-sector R &D, then an increase in $z_{x,L}$ and/or $z_{r,L}$ boosts the incentives to do *L*-specific R &D, thereby increasing the growth rate of the *L*-sector technological knowledge. Until the new steady state, such bias increases the supply of *L*-type intermediate goods, thereby increasing the number of final goods produced with *L*-technology and lowering their relative price concerning the context without government intervention, although in our case with strong removal of scale effects ($\delta =0.8$) the price channel strongly influences the direction of technological-knowledge progress.^28^ and the effect on prices is more intense when there is a direct subsidy to R &D. Hence, in relation to Sc1, $\frac{p_{H}}{p_{L}}$ in (14) increases continuously towards the stable new steady-state level, which implies that *G* is decreasing, but at a falling rate until it reaches its new smaller steady state; however, in relation to the pre-shock steady state, it is observed that $G_{\textrm{Sc2}}^{*}=1.26>G_{\textrm{Sc0}}^{*}=1.24>G_{\textrm{Sc3}}^{*}=1.20$, as depicted in Fig. 1c, d. In other words, since in comparison with Sc1 the exogenous increase in $z_{x,L}$ and/or $z_{r,L}$ induces technological-knowledge bias towards *L*, the stimulus to the relative demand for *L*, arising from the technological-knowledge bias, decreases the *H*-premium, as shown in Table 3: $\left( \frac{w_{H}}{w_{L}}\right) _{\textrm{Sc1}}^{*}=1.28>\left( \frac{w_{H}}{w_{L}}\right) _{\textrm{Sc2}}^{*}=1.16>\left( \frac{w_{H}}{w_{L}}\right) _{\textrm{Sc3}}^{*}=1.13$; that is, the stimulus to the demand for *L*, arising from the technological-knowledge path induced by government policy, increases the *L*-premium. Once in steady state, with a constant technological-knowledge bias, the *L*-premium remains constant as well. In view of the behavior of the technological-knowledge progress, in Sc2 and Sc3 the growth rate is $g_{\textrm{Sc1}}^{*}=1.74{\%}<g_{\textrm{Sc2}}^{*}=1.97{\%}<g_{\textrm{Sc3}}^{*}=2.02{\%}<g_{\textrm{Sc0}}^{*}=2.08{\%}$, the inflation rate is $\pi _{\textrm{Sc1}}^{*}=3.39{\%}>\pi _{\textrm{Sc2}}^{*}=3.04{\%}>\pi _{\textrm{Sc3}}^{*}=2.97{\%}>\pi _{\textrm{Sc0}}^{*}=2.88{\%}$, with a recovery in the welfare level against Sc1. This recovery in welfare is more marked in the private sector. This results also indicate that, with a sufficiently strong increase in government investment in the *L*-sector technological knowledge, the steady-state *H*-premium can return to the level that which prevailed under pre-shock. The consideration of scenarios 2 and 3 also serves to confirm that the intensity of the effects of a policy measure acting directly or indirectly on R &D is distinct and that a policy acting directly on R &D is more effective.

**Table 3:** Pre-COVID-19 and post-COVID-19 steady-state values

Variable	Pre-COVID-19	Post-COVID-19				
	Sc0	Sc1	Sc2	Sc3	Sc4	Sc5
${{\overline{n}}^{*}}$	0.63	0.58	0.62	0.62	0.62	0.61
${G^{*}}$	1.24	1.70	1.26	1.20	1.25	1.22
${W^{*}}$	1.11	1.28	1.16	1.13	1.15	1.13
${g^{*}}$	2.08%	1.74%	1.97%	2.02%	1.75%	1.80%
${\pi ^{*}}$	2.88%	3.39%	3.04%	2.97%	3.38%	3.29%
${Z_{L}^{*}}$	17.76	12.91	16.29	16.92	13.02	13.86
${Z_{H}^{*}}$	33.18	29.24	31.98	32.50	29.33	30.01

Sc4 and Sc5 introduce the possibility of monetary policy conducted by the central bank to assess its effects. Sc4 considers CIA constraints on the production of intermediate goods and on R &D expenditure. As a consequence, and by the same economic mechanisms as in Sc2 and Sc3, the easing of CIAs for the private sector, observed with the changeover from Sc4 to Sc5, leads to a context similar to the Sc3. This means that easing monetary restrictions on the private sector can also be an appropriate policy. As expected, the existence of these restrictions penalizes the growth rate, increases the inflation rate, and harms welfare—compare the results of Sc2 and Sc3 without CIA constraints with those of Sc4 and Sc5 with CIA constraints.

As expected, the numerical results obtained with the five different scenarios are in line with the theoretical results summarized in Table 1.

#### Proposition 3

*The transition dynamics analysis shows that the pre-shock situation can be restored with fiscal and monetary policies and that convergence towards the steady state is guaranteed*.

#### Proof

It results from the numerical resolution of the differential equation (22) for each Scenario. $\square$

### Long-run effects of the productive shock

In addition to the theoretical information in Table 1, this Subsection quantitatively analyzes the intensity and sign of the variation (the percentage change) of the variables of interest in the long run—threshold final good ${\overline{n}}^{*}$ in (24), technological-knowledge bias $G^{*}$ in (25), sector-public premium $W^{*}$ in (26), economic growth rate $g^{*}$ in (27), inflation rate $\pi ^{*}$ in (28), and welfare $Z_{S}^{*}$ in (29)—following a one percent change of the parameters:

- *l* and $\beta _{L}$, to show which parameter has the greatest impact on the variables of interest;
- $z_{x,L}$ and $z_{r,L}$ (fiscal-policy parameters) and $\Omega _{x,L}$ and $\Omega _{r,L}$ (monetary-policy parameters), to reveal which has the greatest impact on shock reversal.

The results of the different elasticities are summarized in Table 4, where $E_{b}^{a^{*}}=\frac{\partial a^{*}}{\partial b}\cdot \frac{b}{a^{*}}$, $a^{*}=\left\{ {\overline{n}}^{*},G^{*},W^{*},g^{*},\pi ^{*},Z_{S}^{*}\right\}$ and $b=\left\{ l,\beta _{L},z_{x,L},z_{r,L},\Omega _{x,L},\Omega _{r,L}\right\}$. As expected, that the signs associated with the different elasticities are aligned with those observed in Table 1.

In the steady-state neighborhood, a permanent increase in either *l* or $\beta _{L}$ of one percentage point, 1 p.p., implies: (i) an increase in ${\overline{n}}^{*}$ of 0.37 p.p.; (ii) a decrease in $G^{*}$, which becomes less accentuated when either *l* or $\beta _{L}$ move away from the steady state, being the response more pronounced at $\beta _{L}$ variations, $\mid E_{\beta _{L}}^{G^{*}}\mid >\mid E_{l}^{G^{*}}\mid$, since it acts directly on R &D activity; (iii) a decrease in $W^{*}$ and $\pi ^{*}$ and the variations are less accentuated as the parameters move away from the value associated with the steady state; (iv) an increase in $g^{*}$ and in the welfare measures, $Z_{L}^{*}$ and $Z_{H}^{*}$. Therefore, a percentage change in these parameters causes a change, in the opposite direction, in the inflation rate as well as in both technological-knowledge gap and wage inequality between sectors, thus favoring the private sector. Moreover, a percentage change in these parameters promotes R &D activities, making them more biased towards the private sector. The promotion of R &D favors the economic growth rate and thus the social welfare measures. The bias towards the private sector makes the improvement of private sector welfare more favorable. The intensity of the effects decreases with the parameter’s value moving away from the steady state, excluding the relationships *l* or $\beta _{L}$ with $Z_{L}^{*}$ in which the intensity of the effects increases, and the relationships *l* or $\beta _{L}$ with ${\overline{n}}^{*}$ in which the intensity of the effects remain stable. In other words, excluding the case $E_{l}^{{\overline{n}}^{*}}=E_{\beta _{L}}^{{\overline{n}}^{*}}=0.37$, there are nonlinearities in the relationships between parameters and variables of interest.

In order to reverse the COVID-19 shock manifested in the fall of *l* and $\beta _{L}$ one should use fiscal policy, increasing $z_{x,L}$ and/or $z_{r,L}$, and/or monetary policy, decreasing $\Omega _{x,L}$ and/or $\Omega _{r,L}$, as shown in the analysis of the results in Table 4. Take the example of the influence on the economic growth rate. Since $E_{l}^{g^{*}}>0,\forall \,l$, and $E_{\beta _{L}}^{g^{*}}>0,\forall \,\beta _{L}$, the decrease of *l* and $\beta _{L}$ decrease the economic growth rate, which can be compensated with an increase of $z_{x,L}$ and/or $z_{r,L}$ since $E_{z_{x,L}}^{g^{*}}>0,\forall \,z_{x,L},$ and $E_{z_{r,L}}^{g^{*}}>0,\forall \,z_{r,L},$ or with a decrease of $\Omega _{x,L}$ and/or $\Omega _{r,L}$ since $E_{\Omega _{x,L}}^{g^{*}}<0,\forall \,\Omega _{x,L},$ and $E_{\Omega _{r,L}}^{g^{*}}<0,\forall \,\Omega _{r,L}$. Indeed, an increase of $z_{x,L}$ and/or $z_{r,L}$ or a decrease of $\Omega _{x,L}$ and/or $\Omega _{r,L}$ generates on the variables of interest an opposite signal effect similar to the shock—with the decrease of *l* and $\beta _{L}$—and, in this case, the evolution of the intensity of the effects as the variation of each policy instruments moves away from the value that gave rise to the steady state are also similar.

#### Proposition 4

*To reverse the COVID-19 shock one should use fiscal and/or monetary policies and with CIAs only in the productive sector*, *fiscal policy is more effective. Regardless of the policy instrument*, *the intensity of the effect on each variable of interest—i.e.*, *the elasticity value—increases as the variation of the instrument moves away from the value that gave rise to the steady state*.

#### Proof

It results from Table 4 that $E_{z_{x,L}}^{a}>E_{\Omega _{x,L}}^{a}$, $E_{z_{r,L}}^{a}>E_{\Omega _{r,L}}^{a}$, $\mid E_{z_{x,L}=b_{1}}^{a}\mid >\mid E_{z_{x,L}=b_{0}}^{a}\mid$, $\mid E_{z_{x,H}=b_{1}}^{a}\mid >\mid E_{z_{x,H}=b_{0}}^{a}\mid$, $\mid E_{z_{r,L}=b_{1}}^{a}\mid >\mid E_{z_{r,L}=b_{0}}^{a}\mid$, $\mid E_{z_{r,H}=b_{1}}^{a}\mid >\mid E_{z_{r,H}=b_{0}}^{a}\mid$, where $a={\overline{n}}^{*},G^{*},W^{*},g^{*},\pi ^{*},Z_{L}^{*},Z_{H}^{*}$ and $b_{1}>b_{0}$. $\square$

It is worth emphasizing some other results, which, in all cases, are aligned with the theoretical and empirical relationships emphasized by the existing literature.

In the steady-state neighborhood, a permanent increase in $\iota$, which, in Table 4, may be seen as equivalent to an increase in $\Omega _{x,L}$ and/or $\Omega _{r,L}$, implies a decrease in $g^{*}$. Therefore, our results are in line with the dominant literature on the relationship between $\iota$ (or $\pi ^{*}$) and $g^{*}$, which, on balance and namely on developed countries, suggests an overwhelming support in favor of negative relationships (e.g., Valdovinos 2003; Akinsola and Odhiambo 2017). Results in Table 4 also show that higher values for $\Omega _{x,L}$ and/or $\Omega _{r,L}$ also imply more negative values of $E_{\Omega _{x,L}}^{g^{*}}$ and $E_{\Omega _{r,L}}^{g^{*}}$, respectively, following a nonlinear trajectory—i.e., there are nonlinearities in the relationships inflation-growth—, such that for higher levels of $\iota$ (through higher $\Omega _{x,L}$ and/or $\Omega _{r,L}$) both elasticities are bigger in module. The economic intuition concerning the economic growth rate is that a rise in inflation lowers the marginal benefit of R &D, inducing entrepreneurs to exit the market. For relatively low inflation, the exit just affects less efficient entrepreneurs and have a small effect on growth. However, if the rate of inflation is high, an additional rise in inflation also affects efficient entrepreneurs, which severely hurts growth. Indeed, high inflation rates are generally considered likely to discourage R &D investments due to their special features, which can make R &D projects highly sensitive to market price distortions, rather than other investment types. A seminal study by Pindyck (1991) showed that increasing uncertainty about future payoffs from R &D projects increases the option value to delay R &D investment decisions. Early research by Fischer (1993), Huizinga (1993), Pindyck and Solimano (1993), and Bruno and Easterly (1998) examined investment behavior under inflation and found a slow investment response to changes in inflation rates as a result of uncertainty in the economic climate. Their results suggested that thus the investors’ preference was to wait for stabilization before investing in R &D.

**Table 4:** Elasticities of the variables of interest with respect to the shock parameters, fiscal-policy parameters, and monetary-policy parameters

Shock parameters	Elasticities, $E_{b}^{a^{*}}=\frac{\partial a^{*}}{\partial b}\cdot \frac{b}{a^{*}}$, where $a^{*}=\left\{ {\overline{n}}^{*},G^{*},W^{*},g^{*},\pi ^{*},Z_{S}^{*}\right\}$ and $b=\left\{ l,\beta _{L}\right\}$						
	$E_{l}^{{\overline{n}}^{*}},E_{\beta _{L}}^{{\overline{n}}^{*}}$	$E_{l}^{G^{*}},E_{\beta _{L}}^{G^{*}}$	$E_{l}^{W^{*}},E_{\beta _{L}}^{W^{*}}$	$E_{l}^{g^{*}},E_{\beta _{L}}^{g^{*}}$	$E_{l}^{\pi ^{*}},E_{\beta _{L}}^{\pi ^{*}}$	$E_{l}^{Z_{L}^{*}},E_{\beta _{L}}^{Z_{L}^{*}}$	$E_{l}^{Z_{H}^{*}},E_{\beta _{L}}^{Z_{L}^{*}}$
$l=0.9$, $\beta _{L}=0.9$	0.37, 0.37	$-$ 0.90, $-$ 1.71	$-$ 0.52, $-$ 0.52	0.84, 0.84	$-$ 0.76, $-$ 0.76	1.46, 1.46	0.58, 0.58
$l=0.8$, $\beta _{L}=0.8$	0.37, 0.37	$-$ 0.80, $-$ 1.44	$-$ 0.51, $-$ 0.51	0.83, 0.83	$-$ 0.63, $-$ 0.63	1.62, 1.62	0.57, 0.57
$l=0.7$, $\beta _{L}=0.7$	0.37, 0.37	$-$ 0.70, $-$ 1.19	$-$ 0.47, $-$ 0.47	0.81, 0.81	$-$ 0.51, $-$ 0.51	1.88, 1.88	0.56, 0.56

Fiscal-policy parameters	Elasticities, $E_{b}^{a^{*}}=\frac{\partial a^{*}}{\partial b}\cdot \frac{b}{a^{*}}$, where $a^{*}=\left\{ {\overline{n}}^{*},G^{*},W^{*},g^{*},\pi ^{*},Z_{S}^{*}\right\}$ and $b=\left\{ z_{x,L},z_{r,L}\right\}$						
	$E_{z_{x,L}}^{{\overline{n}}^{*}},E_{z_{r,L}}^{{\overline{n}}^{*}}$	$E_{z_{x,L}}^{G^{*}},E_{z_{r,L}}^{G^{*}}$	$E_{z_{x,L}}^{W^{*}},E_{z_{r,L}}^{W^{*}}$	$E_{z_{x,L}}^{g^{*}},E_{z_{r,L}}^{g^{*}}$	$E_{z_{x,L}}^{\pi ^{*}},E_{z_{r,L}}^{\pi ^{*}}$	$E_{z_{x,L}}^{Z_{L}^{*}},E_{z_{r,L}}^{Z_{L}^{*}}$	$E_{z_{x,L}}^{Z_{H}^{*}},E_{z_{r,L}}^{Z_{H}^{*}}$
$z_{x,L}=0.1,z_{r,L}=0.1$	0.03, 0.04	$-$ 0.15, $-$ 0.23	$-$ 0.06, $-$ 0.08	0.06, 0.09	$-$ 0.07, $-$ 0.12	0.09, 0.13	0.04, 0.06
$z_{x,L}=0.2,z_{r,L}=0.2$	0.05, 0.07	$-$ 0.35, $-$ 0.56	$-$ 0.12, $-$ 0.17	0.12, 0.18	$-$ 0.18, $-$ 0.30	0.17, 0.24	0.08, 0.12
$z_{x,L}=0.3,z_{r,L}=0.3$	0.08, 0.11	$-$ 0.61, $-$ 1.04	$-$ 0.18, $-$ 0.28	0.19, 0.27	$-$ 0.33, $-$ 0.66	0.25, 0.34	0.13, 0.18

Monetary-policy parameters	Elasticities, $E_{b}^{a^{*}}=\frac{\partial a^{*}}{\partial b}\cdot \frac{b}{a^{*}}$, where $a^{*}=\left\{ {\overline{n}}^{*},G^{*},W^{*},g^{*},\pi ^{*},Z_{S}^{*}\right\}$ and $b=\left\{ \Omega _{x,L},\Omega _{r,L}\right\}$						
	$E_{\Omega _{x,L}}^{{\overline{n}}^{*}},E_{\Omega _{r,L}}^{{\overline{n}}^{*}}$	$E_{\Omega _{x,L}}^{G^{*}},E_{\Omega _{r,L}}^{G^{*}}$	$E_{\Omega _{x,L}}^{W^{*}},E_{\Omega _{r,L}}^{W^{*}}$	$E_{\Omega _{x,L}}^{g^{*}},E_{\Omega _{r,L}}^{g^{*}}$	$E_{\Omega _{x,L}}^{\pi ^{*}},E_{\Omega _{r,L}}^{\pi ^{*}}$	$E_{\Omega _{x,L}}^{Z_{L}^{*}},E_{\Omega _{r,L}}^{Z_{L}^{*}}$	$E_{\Omega _{x,L}}^{Z_{H}^{*}},E_{\Omega _{r,L}}^{Z_{H}^{*}}$
$\Omega _{x,L}=0.2,\Omega _{r,L}=0.2$	$-$ 0.00, $-$ 0.01	0.02, 0.03	0.00, 0.00	$-$ 0.01, $-$ 0.01	0.01, 0.01	$-$ 0.01, $-$ 0.02	$-$ 0.01, $-$ 0.01
$\Omega _{x,L}=0.4,\Omega _{r,L}=0.4$	$-$ 0.01, $-$ 0.01	0.04, 0.06	0.00, 0.01	$-$ 0.02, $-$ 0.03	0.02, 0.03	$-$ 0.03, $-$ 0.04	$-$ 0.01, $-$ 0.02
$\Omega _{x,L}=0.6,\Omega _{r,L}=0.6$	$-$ 0.01, $-$ 0.02	0.06, 0.09	0.01, 0.02	$-$ 0.03, $-$ 0.04	0.03, 0.04	$-$ 0.04, $-$ 0.07	$-$ 0.02, $-$ 0.03

Moreover, since in the steady-state neighborhood, a permanent increase in $\iota$ (through higher $\Omega _{x,L}$ and/or $\Omega _{r,L}$) implies a decrease in the welfare measures, we confirm that there is a nonlinear negative effect of $\iota$ on the steady-state welfare, since the value of the elasticities $E_{\Omega _{x,L}}^{Z_{L}^{*}},$ $E_{\Omega _{r,L}}^{Z_{L}^{*}},$ $E_{\Omega _{x,L}}^{Z_{H}^{*}},$ and $E_{\Omega _{r,L}}^{Z_{H}^{*}}$ decrease at increasing rates with the increase of $\iota$, suggesting that the Friedman rule is optimal. The behavior of the elasticities are consistent with much of the existing literature on welfare costs of inflation, categorized into three areas—CIA studies (e.g., Imrohoroglu 1992; Erosa and Ventura 2002; Camera and Chien 2014), studies using matching models of money (e.g., Molico 2006; Boel and Camera 2009; Chiu and Molico 2010), studies where money plays a precautionary role (e.g., Akyol 2004; Wen 2015)—, which find that, in general, a rise in long-run inflation reduces welfare.

In the steady-state neighborhood, a permanent increase in $\iota$ (through higher $\Omega _{x,L}$ and/or $\Omega _{r,L}$) implies a slight increase in $W^{*}$, contributing to increase wage inequality between public and private sector workers and, therefore, in the country. The model predicts a nonlinear positive influence of $\iota$ on $W^{*}$: the value of the elasticities $E_{\Omega _{x,L}}^{W^{*}}$ and $E_{\Omega _{r,L}}^{W^{*}}$ increase with the increase of $\iota$. The existing theoretical and empirical literature reveals that the relationship between inflation rate and wage inequality is mixed due to multiple channels of influence (e.g., O’Farrell et al. 2016; Amaral 2017). In particular, in line with our result, O’Farrell et al. (2016) conduct a simple theoretical exercise (in a partial equilibrium framework) based on empirical income and wealth distributions and find that the relationship is very small.

## Model with money demand by households and firms

Previously the model emphasized the interaction between fiscal and monetary policy measures in the economy’s productive side. Given its decentralized structure, in monetary terms this has implied an emphasis on the demand for money by firms, while distinguishing between the financing of R &D and the financing of the production of intermediate goods. In this context, a reduced effect of monetary policy on the variables of interest was observed—see the value of elasticities in Table 4, even taking into account relevant values of the parameters associated with the CIAs ($\Omega _{x,L}$ and $\Omega _{r,L}$ equal to 0.2, 0.4 and 0.6)—, which may advise against using this policy in favor of the fiscal policy to combat the COVID-19 shock. In this extension we ask: does this not stem from the abstraction of the model by the demand for money by households, namely induced by liquidity costs/cash operations in consumption and by a specification of money-in-utility in the problem of household optimization? Thus, following, for example, Feenstra (1986), we now consider the households’ maximization problem also with real money balances as arguments of the utility function (e.g., Itaya 1998), being the new discounted lifetime utility maximized subject to the flow budget constraint also modified with pecuniary-transaction costs on consumption (e.g., Lai and Chin 2010, 2013). That is, now we consider that households maximize the discounted lifetime utility

30 $$\begin{aligned} U= \int _{0}^{\infty }\left[ \frac{\left( C(t)^{\chi }m_{C}(t)^{\sigma }\right) ^{1-\theta }}{1-\theta }-\frac{S^{1+\eta }}{1+\eta }\right] e^{-\rho t}\textrm{d}t \end{aligned}$$

subject to $b(t)\le m_{I}(t)$ and

31 $$\begin{aligned} {\dot{a}}(t)+{\dot{m}}(t)&=\left( 1-\tau _{a}\right) \cdot r(t)\cdot a(t)+\sum _{S=L,H}\left( 1-\tau _{w,S}\right) \cdot w_{S}(t)\cdot S\nonumber \\&\quad -\left[ 1+\Phi \left( \frac{C(t)}{m_{C}(t)}\right) ^{\gamma }\right] C(t)+\tau (t)-\pi (t)\cdot m(t)+\iota (t)\cdot b(t) \end{aligned}$$

where a portion of the real money balances, *m*(*t*), can be used for consumption to directly increase households’ utility, $m_{C}(t)=\epsilon \cdot m(t)$, and another portion is directed to finance firms’ investment through the CIA constraints in exchange of a return $\iota (t)-\pi (t)$, $m_{I}(t)=\left( 1-\epsilon \right) \cdot m(t)$; that is, $m(t)=m_{C}(t)+m_{I}(t)$, $\epsilon \in \left( 0,1\right)$. The term $\left[ 1+\Phi \left( \frac{C(t)}{m_{C}(t)}\right) ^{\gamma }\right]$, with $\Phi ,\gamma >0$, is the cost of the cash transaction that households must pay to consume *C* and thus represents the fraction of the households’ real resources that is needed to facilitate the transaction of *C*. Compared to the baseline scenario in Sect. 2.3 the resulting differences arising from solving the problem are (see Appendix D)^29^:

32 $$\begin{aligned} \left( 1-\epsilon \right) \iota&=\left( 1-\tau _{a}\right) r(t)+\pi (t)-\left[ \frac{\sigma }{\chi }\frac{C(t)}{m(t)}+\epsilon \gamma \Phi \left( \frac{C(t)}{m_{C}(t)}\right) ^{\gamma +1}\right. \nonumber \\&\quad \left. +\frac{\sigma }{\chi }\frac{C(t)}{m(t)}\Phi \left( \gamma +1\right) \left( \frac{C(t)}{m_{C}(t)}\right) ^{\gamma }\right] , \end{aligned}$$

which is the new Fisher equation. On the one hand, since holding real money balances also generates a direct utility benefit for the households, measured by the marginal rate of substitution $\frac{\sigma }{\chi }\frac{C(t)}{m(t)}$ in (30), and, on the other hand, since holding real money balances has a direct benefit for the households, measured by the marginal reduction of transaction costs $\epsilon \gamma \Phi \left( \frac{C(t)}{m_{C}(t)}\right) ^{\gamma +1}$ in the flow budget constraint (31), the no-arbitrage condition requires the return to real net financial assets, $\left( 1-\tau _{a}\right) r(t)$, to equal the effective return to real money balances, expressed by $\left( 1-\epsilon \right) \iota +\frac{\sigma }{\chi }\frac{C(t)}{m(t)}+\epsilon \gamma \Phi \left( \frac{C(t)}{m_{C}(t)}\right) ^{\gamma +1}+\frac{\sigma }{\chi }\frac{C(t)}{m(t)}\Phi \left( \gamma +1\right) \left( \frac{C(t)}{m_{C}(t)}\right) ^{\gamma }$. Moreover, since in balanced growth path $\frac{{\dot{C}}}{C}=g_{C}=g_{m}=g_{m_{C}}=g_{m_{I}}=g$ the long-run optimal path is now given by

33 $$\begin{aligned} g^{*} =\frac{\left( 1-\tau _{a}\right) r^{*}-\rho }{1-\left( \chi +\sigma \right) \left( 1-\theta \right) } \end{aligned}$$

where: (i) the condition $(\chi +\sigma )\cdot (1-\theta )<1$ is required to have $g^{*}>0$; (ii) from (20) and (33), the stable and unique steady-state interest rate is:

34 $$\begin{aligned} r^{*}=\frac{\frac{\beta _{L}}{\varsigma _{L}}\cdot \left( \frac{1-z_{x,L}+\Omega _{x,L}\cdot \iota }{1-z_{r,L}+\Omega _{r,L}\cdot \iota }\right) \left( \frac{q-1}{q}\right) \cdot l\cdot L^{1-\delta }\left[ \frac{p_{L}^{*}\left( 1-\alpha \right) }{1-z_{x,L}+\Omega _{x,L}\cdot \iota }\right] ^{\frac{1}{\alpha }}\left( q^{\frac{1-\alpha }{\alpha }}-1\right) \left[ 1-\left( \chi +\sigma \right) \left( 1-\theta \right) \right] +\rho }{\left( q^{\frac{1-\alpha }{\alpha }}-1\right) \left[ 1-\left( \chi +\sigma \right) \left( 1-\theta \right) \right] +\left( 1-\tau _{a}\right) }, \end{aligned}$$

where $p_{L}^{*}$ can be determined by the expressions (15) and (24); i.e., $p_{L}^{*}={\exp (-\alpha )}\cdot {{\overline{n}}^{*-\alpha }}$. Hence, for a given $\theta$, the households’ willingness to smooth out consumption over time is dampened by the elasticity of utility with respect to consumption and with respect to real money balances. It also results that (33) fell into (10) in the particular case of an utility function in (30) with constant returns to scale in *C* and *m*; i.e., $\chi +\sigma =1$.

Equation (32) can be naturally written to the balanced growth path:

35 $$\begin{aligned} \pi ^{*}&=\left( 1-\epsilon \right) \iota -\left[ 1-\left( \chi +\sigma \right) \left( 1-\theta \right) \right] g^{*}-\rho \nonumber \\&\quad +\left[ \frac{\sigma }{\chi }\frac{C^{*}}{m^{*}}+\epsilon \gamma \Phi \left( \frac{C^{*}}{m_{C}^{*}}\right) ^{\gamma +1}+\frac{\sigma }{\chi }\frac{C^{*}}{m^{*}}\Phi \left( \gamma +1\right) \left( \frac{C^{*}}{m_{C}^{*}}\right) ^{\gamma }\right] , \end{aligned}$$

where: $g^{*}$ and $r^{*}$ are given by (33) and (34), respectively. To know the sign of the relationship between $\iota$ and $\pi$ it is necessary to determine the sign of the $\iota$ impact in $C^{*}$. Indeed, from $m_{I}=\left( 1-\epsilon \right) \cdot m$ and $m_{C}=\epsilon \cdot m$, we have that $m_{C}=\frac{\epsilon }{1-\epsilon }m_{I}$, $\frac{C}{m}=\frac{1}{1-\epsilon }\cdot \frac{C}{m_{I}}$ and $\frac{C}{m_{C}}=\frac{1}{\epsilon }\frac{1}{1-\epsilon }\cdot \frac{C}{m_{I}}$, where $m_{I}=b=\sum _{S=L,H}\Omega _{x,S}\cdot X_{S}+\sum _{S=L,H}\Omega _{r,S}\cdot R_{S}$ (see Sect. 3.3). In turn, taking also into account (39), Eq. (40) is now given by

36 $$\begin{aligned} \left[ 1+\Phi \left( \frac{1-\epsilon }{\epsilon }\frac{C^{*}}{b}\right) ^{\gamma }\right] C^{*}&=\left( 1-\tau _{a}\right) \cdot r^{*}+\sum _{S=L,H}\left( 1-\tau _{w,S}\right) \cdot w_{S}\cdot S\nonumber \\&\quad +b\cdot \iota +\left( q^{\frac{1-\alpha }{\alpha }}-1\right) \sum _{S=L,H}\left( 1-z_{r,S}+\Omega _{r,S}\cdot \iota \right) \cdot R_{S}. \end{aligned}$$

Since this term increases in $\iota$, then $\frac{C}{m}$, $\frac{C}{m_{I}}$, and $\frac{C}{m_{C}}$ also increase in $\iota$ to satisfy (36). Hence,

### Proposition 5

*The long-run inflation rate*, $\pi ^{*}$, *remains as an increasing function of the exogenous monetary policy variable*, $\iota$.

### Proof

It results directly from (32), which, considering (33), can be written as $\pi ^{*}=\left( 1-\epsilon \right) \iota -\left[ 1-\left( \chi +\sigma \right) \left( 1-\theta \right) \right] g^{*}-\rho +\left[ \frac{\sigma }{\chi }\frac{C^{*}}{m^{*}}+\epsilon \gamma \Phi \left( \frac{C^{*}}{m_{C}^{*}}\right) ^{\gamma +1}+\frac{\sigma }{\chi }\frac{C^{*}}{m^{*}}\Phi \left( \gamma +1\right) \left( \frac{C^{*}}{m_{C}^{*}}\right) ^{\gamma }\right]$ and the partial derivative of $g^{*}$ with respect to $\iota$ is negative—see Appendix E. $\square$

In general, results from this extension are summarized in Proposition 6:

### Proposition 6

*Considering the function money-in-utility function and pecuniary-transaction costs on consumption*, *it is observed that*, *given a change in one of the monetary-policy parameters* $\Omega _{x,S}$ *or* $\Omega _{r,S}$, $S=\left\{ L,H\right\}$: (A) *the sign and the intensity of the effect on the variables of interest* ${\overline{n}}^{*}$, $G^{*}$ *and* $W^{*}$ *is not affected*; (B) *the sign of the effect on the variables of interest* $g^{*}$, $\pi ^{*}$, *and* $Z_{S}^{*}$ *remains unchanged*, *but the intensity is affected*.

### Proof

In this new situation, the steady-state expressions for the threshold final good in (24), the technological-knowledge bias in (25), and the sector-public premium in (26) are not affected. Therefore, the derivatives $\frac{\partial {\overline{n}}^{*}}{\partial \Omega _{x,S}}$, $\frac{\partial {\overline{n}}^{*}}{\partial \Omega _{r,S}}$, $\frac{\partial G^{*}}{\partial \Omega _{x,S}}$, $\frac{\partial G^{*}}{\partial \Omega _{r,S}}$, $\frac{\partial W^{*}}{\partial \Omega _{x,S}}$, and $\frac{\partial W^{*}}{\partial \Omega _{r,S}}$ do not change because the expressions remain unchanged. In turn, the steady-state expression for the economic growth rate in (33), the inflation rate in (32), and the welfare measures in (29) are affected—the latter due to the change of the expression to $g^{*}$. Hence, the sign of each derivative $\frac{\partial g^{*}}{\partial \Omega _{x,S}}$, $\frac{\partial g^{*}}{\partial \Omega _{r,S}}$, $\frac{\partial \pi ^{*}}{\partial \Omega _{x,S}}$, $\frac{\partial \pi ^{*}}{\partial \Omega _{r,S}}$, $\frac{\partial Z_{S}^{*}}{\partial \Omega _{x,S}}$, and $\frac{\partial Z_{S}^{*}}{\partial \Omega _{r,S}}$ remains unchanged, its value is now different. $\square$

It can therefore be stated that including in the DTC model developed the real money balances, as an argument of the utility function, and monetary-transaction costs, the results are similar to those that would be obtained with a traditional endogenous growth model. In fact, this extension has no implications for the final good threshold ${\overline{n}}^{*}$, the technological-knowledge bias $G^{*}$, and the public-sector premium $W^{*}$, which result from the use of the DTC model. This extension has implications for the steady-state economic growth rate $g^{*}$ and inflation rate $\pi ^{*}$, as it would in a traditional endogenous growth model. We can however state that, because we consider the DTC model, the welfare functions in the public and private sectors are affected by the extension, since the growth rate is affected.

## Concluding remarks

We have proposed an endogenous growth model where individuals decide consumption, labor-level supplied and savings and where two productive sectors of perfectly competitive final goods are considered. The private sector combines specific labor with a specific set of (complementary) quality-adjusted intermediate goods and the public sector uses specific labor complemented with a continuum of specific quality-adjusted intermediate goods. Labor levels are corrected for their productivity and, due to the COVID-19 pandemic, there is a negative shock to private sector labor productivity. Intermediate goods, which are improved in the R &D sector, are produced in monopolistic competition and the R &D technology in the private sector is also supposed to suffer from the pandemic. Hence, the direction of the technological knowledge, which, in line with the DTC mechanism, drives the wage inequality between sectors, is influenced by the dynamics of the economic system, by the pandemic as well as by fiscal and monetary policies conducted, respectively, by the government and the central bank. The former policy is materialized in subsidies for the production of intermediate goods or the production of innovations, while the latter policy is materialized in the existence of CIA constraints.

Concerning wage inequality between sectors, we find that a negative shock in labor level productivity in the private sector causes an immediate steep drop in the private premium. Indeed, the private sector loses competitiveness, the technological-knowledge progress benefits the public sector, the relative private wage falls, the inflation rate increases, and both the economic growth rate and the welfare decline. This immediate effect can be reverted in the transition dynamics towards the constant steady-state wage premium, due to the stimulus to the demand for labor in private sector resulting from the technological-knowledge bias, which is also affected by the shock in private R &D technology. With an adequate fiscal policy, whether or not combined with monetary policy, the steady-state pre-shock wage premium can be ensured, and the economic growth rate, the inflation rate and the level of welfare. The impact of monetary policy on economic growth, inflation and welfare can be affected by introducing demand for money by households in the model, namely induced by pecuniary-transaction costs on consumption and by money-in-utility function.

Our framework is still quite stylized as we deal with only one country, no horizontal R &D, and only follower firms that support R &D. This encourages extensions in several directions. For example, with two or more countries, both intra- and inter-country wage inequality between sectors can be analyzed and under different international trade regimes. In future research, we also intend to explore how endogenous human capital accumulation reacts to government intervention and affects wage inequality between sectors. Moreover, considering that some recent empirical literature points to a decrease in labor share, it would be interesting to make a detailed analysis on the impact of the fiscal and monetary policies in this context.

## Data Availability

The data that support the findings of this study are openly available in The Organisation for Economic Co-operation and Development (OECD) and Eurostat.

## Appendix A

From the definition of price indexes, ${p_{L}=p_{n}}\cdot {\left( 1-n\right) ^{\alpha }}$ and ${p_{H}=p_{n}}\cdot {n^{\alpha }}$, we have that: (i) $\frac{p_{H}}{p_{L}}$ is a continuous function of *n*; (ii) $\frac{p_{H}}{p_{L}}$ is assumed to be a positive constant; (iii) $\frac{p_{H}}{p_{L}}$ varies negatively with *n*, (iv) $\lim _{n\rightarrow 1}\frac{p_{H}}{p_{L}}=0$, and (v) $\lim _{n\rightarrow 0}\frac{p_{H}}{p_{L}}=\infty$. Using (i)–(v) by the Intermediate Value Theorem there is a ${\overline{n}}\in \left[ 0,1\right]$. Since the output of each variety *n* is produced in perfect competition, firms opt for producing *n* with the lowest price. Therefore, for $n={\overline{n}}$ they are indifferent between labor types and thus $\frac{p_{H}}{p_{L}}=\left( \frac{{\overline{n}}}{1-{\overline{n}}}\right) ^{\alpha }$, but for $n<{\overline{n}}$ $\left( n>{\overline{n}}\right)$ they choose *L* $\left( H\right)$. Bearing in mind the definition of price indexes and the condition that characterizes the threshold final good, we can determine the following expression for the threshold final good ${\overline{n}}=\left[ 1+\left( \frac{p_{H}}{p_{L}}\right) ^{\frac{1}{\alpha }}\right] ^{-1}.$

We now only need to determine the expression for $\frac{p_{H}}{p_{L}}$. Considering that the value of each final good produced in country *S* is constant for all *n*, we have that at each time period (i) $p_{n}Y_{n}=\left( p_{L}\right) ^{\frac{1}{\alpha }}\left( \frac{1-\alpha }{p(j,k,t)}\right) ^{\frac{1-\alpha }{\alpha }}Q_{L}\cdot l\cdot L_{n}$ for $S=L$ and $p_{n}Y_{n}=\left( p_{H}\right) ^{\frac{1}{\alpha }}\left( \frac{1-\alpha }{p(j,k,t)}\right) ^{\frac{1-\alpha }{\alpha }}Q_{H}\cdot h\cdot H_{n}$ for $S=H$, which, since the terms are constants with respect to *n*, implies necessarily that $L_{n}$ and $H_{n}$ are also constants that are defined as $L_{n}=\frac{L}{{\overline{n}}}$ and $H_{n}=\frac{H}{1-{\overline{n}}}$ as a result of considering that $L=\int _{0}^{{\overline{n}}}L_{n}\textrm{d}n$ and $H=\int _{{\overline{n}}}^{1}H_{n}\textrm{d}n$; (ii) $\underbrace{p_{n}Y_{n}}_{S=L}=\underbrace{p_{n}Y_{n}}_{S=H}$ from where we derive that $\frac{p_{H}}{p_{L}}=\left( \frac{Q_{H}}{Q_{L}}\frac{h\cdot H}{l\cdot L}\frac{{\overline{n}}}{1-{\overline{n}}}\right) ^{\alpha }$. Replacing this expression in the previous we obtain ${\overline{n}}=\left[ 1+\left( \frac{Q_{H}}{Q_{L}}\frac{h\cdot H}{l\cdot L}\right) ^{\frac{1}{2}}\right] ^{-1}=\left[ 1+\left( G\frac{h\cdot H}{l\cdot L}\right) ^{\frac{1}{2}}\right] ^{-1}$ in (13).

## Appendix B

This appendix proves that the model is consistent, ensuring that is in fact a dynamic general-equilibrium endogenous growth model: the aggregate final good, *Y*, is used in consumption, *C*, and investment, $X+R$; firms and households are rational and solve their problems; free-entry R &D conditions are met; and markets clear. Taking into account that the equilibrium sector-specific probability of successful R &D is independent of the quality rung—see (20); i.e., $I_{S}(j,k,t)=I_{S}(j,t),\;\forall k\in {\mathbb {N}}$ and $S=L,H$—we have, from the definition of market value of a firm, that $V_{S}(j,k,t)=\frac{\Pi _{S}(j,k,t)}{r(t)+I_{S}(j,k-1,t)}$ and thus

37 $$\begin{aligned} r(t)\cdot V_{S}(j,k,t)=\left( q-1\right) \cdot \left( 1-z_{x,S}+\Omega _{x,S}\cdot \iota \right) \cdot x_{S}\left( j,k,t\right) -q^{\frac{\alpha -1}{\alpha }}\cdot y_{S}(j,k,t), \end{aligned}$$

where: $x_{L}\left( j,k,t\right) =\int _{0}^{{\overline{n}}}x_{n}(j,k,t)\cdot \textrm{d}n$, $x_{H}\left( j,k,t\right) =\int _{{\overline{n}}}^{1}x_{n}(j,k,t)\cdot \textrm{d}n$, $\Pi _{S}(j,k,t)=\left( q-1\right) \left( 1-z_{x,S}+\Omega _{x,S}\cdot \iota \right) x_{S}\left( j,k,t\right)$, and $I_{S}(j,t)\cdot V_{S}(j,k,t)=q^{\frac{\alpha -1}{\alpha }}\cdot y_{S}(j,k,t)$.^30^ By integrating (37) over *j*,^31^ we have: $r\cdot a_{S}=p_{S}\left( 1-\alpha \right) Y_{S}-\left( 1-z_{x,S}+\Omega _{x,S}\cdot \iota \right) X_{S}-q^{(\frac{\alpha -1}{\alpha })}(1-z_{r,S}+\Omega _{r,S}\cdot \iota )R_{S}$, where, by definition, $a_{L}=\int _{0}^{J}V_{L}(j,k)\cdot \textrm{d}j$ and $a_{H}=\int _{J}^{1}V_{H}(j,k)\cdot \textrm{d}j$ are the market value of all the firms that produce intermediate goods *j* at time *t* complementary with sectors *L* and *H*, respectively, $X_{L}=\int _{0}^{J}x_{L}(j,k\cdot \textrm{d}j$, $X_{H}=\int _{J}^{1}x_{H}(j,k)\cdot \textrm{d}j$, $R_{L}=\int _{0}^{J}y_{L}(j,k)\cdot \textrm{d}j$, $R_{H}=\int _{J}^{1}y_{H}(j,k)\cdot \textrm{d}j$, and $q\cdot \left( 1-z_{x,S}+\Omega _{x,S}\cdot \iota \right) \cdot X_{S}=\left( 1-\alpha \right) \cdot p_{S}\cdot Y_{S}$. In turn, since $a=\sum _{S=L,H}a_{S}$ results

38 $$\begin{aligned} r\cdot a&=\left( 1-\alpha \right) Y-X+\sum _{S=L,H}\left( z_{x,S}-\Omega _{x,S}\cdot \iota \right) X_{S}\nonumber \\&\quad -q^{(\frac{\alpha -1}{\alpha })}\sum _{S=L,H}(1-z_{r,S}+\Omega _{r,S}\cdot \iota )R_{S}, \end{aligned}$$

where $Y=\sum _{S=L,H}p_{S}Y_{S}$ and $X=\sum _{S=L,H}X_{S}$. Still from the definition of market value of a firm, $V_{S}(j,k,t)=\frac{\Pi _{S}(j,k,t)}{r(t)+I_{S}(j,t)}$, but given the expression for profits, $\Pi _{S}(j,k,t)$, and the expression for the equilibrium probability, $I_{S}(j,t)$ (e.g., in large brackets of (20) for $I_{L}(j,t)$), it results that $V_{S}(j,k,t)=\frac{\varsigma _{S}}{\beta _{S}}\cdot q^{(\frac{\alpha -1}{\alpha })}\cdot \left( 1-z_{r,S}+\Omega _{r,S}\cdot \iota \right) \cdot S^{\delta }\cdot q^{k(j,t)(\frac{1-\alpha }{\alpha })}$; hence, $a_{L}=\int _{0}^{J}V_{L}(j,k)\cdot \textrm{d}j=\frac{\varsigma _{L}}{\beta _{L}}\cdot q^{(\frac{\alpha -1}{\alpha })}\cdot \left( 1-z_{r,L}+\Omega _{r,L}\cdot \iota \right) \cdot L^{\delta }\cdot Q_{L}$ and $a_{H}=\int _{J}^{1}V_{H}(j,k)\cdot \textrm{d}j=\frac{\varsigma _{H}}{\beta _{H}}\cdot q^{(\frac{\alpha -1}{\alpha })}\cdot \left( 1-z_{r,H}+\Omega _{r,H}\cdot \iota \right) \cdot H^{\delta }\cdot Q_{H}$ and, bearing in mind time derivatives as well as (21), it results that ${\dot{a}}_{S}=\left( 1-z_{r,S}+\Omega _{r,S}\cdot \iota \right) \cdot \left( 1-q^{\frac{1-\alpha }{\alpha }}\right) \cdot R_{S}$ since ${\dot{Q}}_{S}=I_{S}(j,t)\cdot \left( q^{\frac{1-\alpha }{\alpha }}-1\right) \cdot Q_{S}$; therefore,

39 $$\begin{aligned} {\dot{a}}=\left( 1-q^{\frac{1-\alpha }{\alpha }}\right) \sum _{S=L,H}\left( 1-z_{r,S}+\Omega _{r,S}\cdot \iota \right) \cdot R_{S}, \end{aligned}$$

where: ${\dot{a}}=\sum _{S=L,H}{\dot{a}}_{S}$. On the other hand, since $\tau (t)={\dot{m}}(t)+\pi (t)\cdot m(t)$, we can write from (8) that

40 $$\begin{aligned} {\dot{a}}=r\cdot a-\tau _{a}\cdot r\cdot a+\sum _{S=L,H}w_{S}\cdot S-\sum _{S=L,H}\tau _{w,S}\cdot w_{S}\cdot S-C+b\cdot \iota , \end{aligned}$$

where: $a(t)=\sum _{S=L,H}a_{S}(t)$. Replacing (38) and (39) in (40), and taking into account (12), $\sum _{S=L,H}w_{S}\cdot S=\alpha \cdot Y$ since $w_{S}=\frac{\alpha \cdot p_{S}\cdot Y_{S}}{S}$, and the amount of money lent by households $b=\sum _{S=L,H}\Omega _{x,S}\cdot X_{S}+\sum _{S=L,H}\Omega _{r,S}\cdot R_{S}$, we have:

41 $$\begin{aligned} Y=C+X+R. \end{aligned}$$

That is, resources, *Y* in (1), that are not consumed, *C* in Sect. 2.3, are indeed used in the production of intermediates, *X* in (16), and in the R &D sector, *R* in (21).

## Appendix C

Regarding the proof of Proposition 2, since ${p_{L}^{*}}={\exp (-\alpha )}\cdot {{\overline{n}}^{*-\alpha }}$ we have from (24) that

42 $$\begin{aligned}&p_{L}^{*} \left[ {1}+\frac{\beta _{H}}{\beta _{L}}\cdot \frac{\varsigma _{L}}{\varsigma _{H}}\cdot \left( \frac{1-z_{r,L}+\Omega _{r,L}\cdot \iota }{1-z_{r,H}+\Omega _{r,H}\cdot \iota }\right) \right. \nonumber \\&\quad \cdot \left. \left( \frac{1-z_{x,L}+\Omega _{x,L}\cdot \iota }{1-z_{x,H}+\Omega _{x,H}\cdot \iota }\right) ^{\frac{1-\alpha }{\alpha }}\cdot \frac{h}{l}\cdot \left( \frac{H}{L}\right) ^{1-\delta }\right] ^{\alpha }\text {.} \end{aligned}$$

Substituting (42) in (27) and rearranging the terms, yields

43 $$\begin{aligned} g^{*}&=\frac{\left( 1-\tau _{a}\right) \cdot \frac{\beta _{L}}{\varsigma _{L}}\cdot \left( \frac{1-z_{x,L}+\Omega _{x,L}\cdot \iota }{1-z_{r,L}+\Omega _{r,L}\cdot \iota }\right) \left( \frac{q-1}{q}\right) \cdot l\cdot L^{1-\delta }\left[ \frac{p_{L}^{*}\left( 1-\alpha \right) }{1-z_{x,L}+\Omega _{x,L}\cdot \iota }\right] ^{\frac{1}{\alpha }}-\rho }{\left( q^{\frac{1-\alpha }{\alpha }}-1\right) ^{-1}+\theta }\nonumber \\&=\frac{\left( 1-\tau _{a}\right) \cdot \frac{\beta _{L}}{\varsigma _{L}}\cdot \left( \frac{q-1}{q}\right) \cdot l\cdot L^{1-\delta }\left( 1-\alpha \right) ^{\frac{1}{\alpha }}}{\left( q^{\frac{1-\alpha }{\alpha }}-1\right) ^{-1}+\theta }\left\{ \frac{\left( 1-z_{x,L}+\Omega _{x,L}\cdot \iota \right) ^{1-\frac{1}{\alpha }}}{1-z_{r,L}+\Omega _{r,L}\cdot \iota }\left( p_{L}^{*}\right) ^{\frac{1}{\alpha }}\right\} \nonumber \\&\quad -\frac{\rho }{\left( q^{\frac{1-\alpha }{\alpha }}-1\right) ^{-1}+\theta }\nonumber \\&=\frac{\left( 1-\tau _{a}\right) \cdot \frac{\beta _{L}}{\varsigma _{L}}\cdot \left( \frac{q-1}{q}\right) \cdot l\cdot L^{1-\delta }\left( 1-\alpha \right) ^{\frac{1}{\alpha }}}{\left( q^{\frac{1-\alpha }{\alpha }}-1\right) ^{-1}+\theta }\cdot \exp (-1)\nonumber \\&\quad \times \left\{ \frac{\left( 1-z_{x,L}+\Omega _{x,L}\cdot \iota \right) ^{1-\frac{1}{\alpha }}}{1-z_{r,L}+\Omega _{r,L}\cdot \iota }+\frac{\beta _{H}}{\beta _{L}}\right. \nonumber \\&\quad \left. \cdot \frac{\varsigma _{L}}{\varsigma _{H}}\cdot \frac{\left( 1-z_{x,H}+\Omega _{x,H}\cdot \iota \right) ^{1-\frac{1}{\alpha }}}{1-z_{r,H}+\Omega _{r,H}\cdot \iota }\cdot \frac{h}{l}\cdot \left( \frac{H}{L}\right) ^{1-\delta }\right\} \nonumber \\&\quad -\frac{\rho }{\left( q^{\frac{1-\alpha }{\alpha }}-1\right) ^{-1}+\theta }\text {.} \end{aligned}$$

Then, notice that the sign of $\frac{\partial g^{*}}{\partial \iota }$ is determined by the expression inside the brackets in (43), and that

$$\begin{aligned} \frac{\partial }{\partial \iota }\left[ \frac{\left( 1-z_{x,S}+\Omega _{x,S}\cdot \iota \right) ^{1-\frac{1}{\alpha }}}{1-z_{r,S}+\Omega _{r,S}\cdot \iota }\right]&=-\left( \frac{1-\alpha }{\alpha }\right) \Omega _{x,S}\frac{\left( 1-z_{x,S}+\Omega _{x,S}\cdot \iota \right) ^{-\frac{1}{\alpha }}}{1-z_{r,S}+\Omega _{r,S}\cdot \iota }\\&\quad -\Omega _{r,S}\frac{\left( 1-z_{x,S}+\Omega _{x,S}\cdot \iota \right) ^{1-\frac{1}{\alpha }}}{\left( 1-z_{r,S}+\Omega _{r,S}\cdot \iota \right) ^{2}}\\&<0,\quad \forall \,S\,\in \left\{ L,H\right\} , \end{aligned}$$

which implies $\frac{\partial g^{*}}{\partial \iota }<0$. Consequently,

$$\begin{aligned} \frac{\partial \pi ^{*}}{\partial \iota } =1-\theta \cdot \frac{\partial g^{*}}{\partial \iota }>0. \end{aligned}$$

## Appendix D

From the standard Optimal Control Theory, we consider the auxiliary Hamiltonian function, assuming the (static) CIA constraint is binding as in the baseline model; i.e., $b(t)=m_{I}(t)$,

$$\begin{aligned} H&=\left[ \frac{\left( C(t)^{\chi }m_{C}(t)^{\sigma }\right) ^{1-\theta }}{1-\theta }-\frac{S^{1+\eta }}{1+\eta }\right] e^{-\rho t}+\lambda (t)\left[ b(t)-m_{I}(t)\right] \\&\quad +\mu (t)\left\{ \left( 1-\tau _{a}\right) \cdot r(t)\cdot a(t)+\sum _{S=L,H}\left( 1-\tau _{w,S}\right) \cdot w_{S}(t)\cdot S\right. \\&\quad -\left[ 1+\Phi \left( \frac{C(t)}{m_{C}(t)}\right) ^{\gamma }\right] C(t)\\&\quad \left. +\tau (t)-\pi (t)\cdot m(t)+\iota (t)\cdot b(t)\right\} \end{aligned}$$

where: *a* and *m* are the state variables; $\lambda$ and $\mu$ are the costate variables; *C*, *S* and *b* are the control variables. Then, the necessary conditions under the Maximum Principle are:

44 $$\begin{aligned} \frac{\partial H}{\partial C(t)} =0\Leftrightarrow \chi \left[ \frac{C(t)^{\chi }m_{C}(t)^{\sigma }}{C(t)}\right] ^{1-\theta }e^{-\rho t}-\mu (t)\Phi \left( \gamma +1\right) \left( \frac{C(t)}{m_{C}(t)}\right) ^{\gamma }-\mu (t)=0 \end{aligned}$$

45 $$\begin{aligned} \frac{\partial H}{\partial b(t)} =0\Leftrightarrow \lambda (t)+\mu (t)\cdot \iota =0 \end{aligned}$$

46 $$\begin{aligned} \frac{\partial H}{\partial S(t)} =0\Leftrightarrow S^{\eta }\cdot e^{-\rho t}+\mu (t)\cdot \left( 1-\tau _{w,S}\right) \cdot w_{S}(t)=0 \end{aligned}$$

47 $$\begin{aligned} \frac{\partial H}{\partial a(t)} =-{\dot{\mu }}(t)\Leftrightarrow \left( 1-\tau _{a}\right) r(t)+\frac{{\dot{\mu }}(t)}{\mu (t)}=0 \end{aligned}$$

48 $$\begin{aligned} \frac{\partial H}{\partial m(t)}&=-{\dot{\mu }}(t)\Leftrightarrow \sigma \left[ \frac{C(t)^{\chi }m_{C}(t)^{\sigma }}{m(t)}\right] ^{1-\theta }e^{-\rho t}+\mu (t)\epsilon \gamma \Phi \left( \frac{C(t)}{m_{C}(t)}\right) ^{\gamma +1}\nonumber \\&\quad -\mu (t)\pi (t)-\left( 1-\epsilon \right) \lambda (t)+{\dot{\mu }}(t)=0 \end{aligned}$$

49 $$\begin{aligned} \frac{\partial H}{\partial \lambda (t)} =0 \end{aligned}$$

50 $$\begin{aligned} \frac{\partial H}{\partial \mu (t)} ={\dot{a}}(t)+{\dot{m}}(t) \end{aligned}$$

Rearranging (44) we have ${\frac{C(t)}{\chi }\left[ 1+\Phi \left( \gamma +1\right) \left( \frac{C(t)}{m_{C}(t)}\right) ^{\gamma }\right] \mu (t)=\left[ C(t)^{\chi }m_{C}(t)^{\sigma }\right] ^{1-\theta }e^{-\rho t}}$, which, used together with (45), in (48) gives:

$$\begin{aligned} \frac{{\dot{\mu }}(t)}{\mu (t)}&=\pi (t)-\left( 1-\epsilon \right) \iota -\epsilon \gamma \Phi \left( \frac{C(t)}{m_{C}(t)}\right) ^{\gamma +1}\\&\quad -\frac{C(t)}{m(t)}\frac{\sigma }{\zeta }\left[ 1+\Phi \left( \gamma +1\right) \left( \frac{C(t)}{m_{C}(t)}\right) ^{\gamma }\right] . \end{aligned}$$

Bearing in mind this result and (47) we have the new Fisher equation (32):

51 $$\begin{aligned} \left( 1-\epsilon \right) \iota&=\left( 1-\tau _{a}\right) r(t)+\pi (t)-\epsilon \gamma \Phi \left( \frac{C(t)}{m_{C}(t)}\right) ^{\gamma +1}\nonumber \\&\quad -\frac{C(t)}{m(t)}\frac{\sigma }{\chi }\left[ 1+\Psi \left( \gamma +1\right) \left( \frac{C(t)}{m_{C}(t)}\right) ^{\gamma }\right] \end{aligned}$$

Now, we define a new variable $Q(t)=1+\Phi \left( \gamma +1\right) \left( \frac{C(t)}{m_{C}(t)}\right) ^{\gamma }$ and rewrite (44) as $\mu (t)Q(t)=\chi C(t)^{\chi \left( 1-\theta \right) -1}m_{C}(t)^{\sigma \left( 1-\theta \right) }e^{-\rho t}$. Then, log-differentiating this result with respect to time we have:

$$\begin{aligned} \frac{{\dot{\mu }}(t)}{\mu (t)} =\left[ \chi \left( 1-\theta \right) -1\right] \frac{{\dot{C}}(t)}{C(t)}+\sigma \left( 1-\theta \right) \frac{{\dot{m}}_{C}(t)}{m_{C}(t)}-\rho -\frac{{\dot{Q}}(t)}{Q(t)}. \end{aligned}$$

Finally, since in the balanced growth path $\frac{{\dot{C}}(t)}{C(t)}=\frac{{\dot{m}}(t)}{m(t)}=\frac{\dot{m_{C}}(t)}{m_{C}(t)}$, that also implies $\frac{{\dot{Q}}(t)}{Q(t)}=0$, and (47) in this result will give the new Euler equation for consumption (33):

52 $$\begin{aligned} \frac{{\dot{C}}(t)}{C(t)} =\frac{\left( 1-\tau _{a}\right) r(t)-\rho }{1-\left( \chi +\sigma \right) \left( 1-\theta \right) }. \end{aligned}$$

Moreover, dividing (46) across different types of workers, i.e., *H* over *L*, we find (11). The transversality conditions are again $\lim _{t\rightarrow \infty }\mu (t)a(t)=0\;\text {and}\;\lim _{t\rightarrow \infty }\mu (t)m(t)=0$.

## Appendix E

Regarding the proof of Proposition 5, substituting (34) and (24) in (33), and rearranging the terms, we find

53 $$\begin{aligned} g^{*}&=\frac{\left( 1-\tau _{a}\right) \cdot \frac{\beta _{L}}{\varsigma _{L}}\cdot l\cdot L^{1-\delta }\cdot \left( 1-\alpha \right) ^{\frac{1}{\alpha }}\cdot \left( q^{\frac{1-\alpha }{\alpha }}-1\right) \cdot \left( \frac{q-1}{q}\right) \cdot \left[ 1-\left( \chi +\sigma \right) \left( 1-\theta \right) \right] }{\left[ 1-\left( \chi +\sigma \right) \left( 1-\theta \right) \right] \cdot \left\{ \left( q^{\frac{1-\alpha }{\alpha }}-1\right) \left[ 1-\left( \chi +\sigma \right) \left( 1-\theta \right) \right] +\left( 1-\tau _{a}\right) \right\} }\nonumber \\&\quad \left\{ \frac{\left( 1-z_{x,L}+\Omega _{x,L}\cdot \iota \right) ^{1-\frac{1}{\alpha }}}{1-z_{r,L}+\Omega _{r,L}\cdot \iota }\cdot \left( p_{L}^{*}\right) ^{\frac{1}{\alpha }}\right\} \nonumber \\&\quad +\frac{\rho }{\left[ 1-\left( \chi +\sigma \right) \left( 1-\theta \right) \right] \cdot \left\{ \left( q^{\frac{1-\alpha }{\alpha }}-1\right) \left[ 1-\left( \chi +\sigma \right) \left( 1-\theta \right) \right] +\left( 1-\tau _{a}\right) \right\} }\nonumber \\&\quad -\frac{-\rho }{1-\left( \chi +\sigma \right) \left( 1-\theta \right) }\nonumber \\&=\frac{\left( 1-\tau _{a}\right) \cdot \frac{\beta _{L}}{\varsigma _{L}}\cdot l\cdot L^{1-\delta }\cdot \left( 1-\alpha \right) ^{\frac{1}{\alpha }}\cdot \left( q^{\frac{1-\alpha }{\alpha }}-1\right) \cdot \left( \frac{q-1}{q}\right) \cdot \left[ 1-\left( \chi +\sigma \right) \left( 1-\theta \right) \right] }{\left[ 1-\left( \chi +\sigma \right) \left( 1-\theta \right) \right] \cdot \left\{ \left( q^{\frac{1-\alpha }{\alpha }}-1\right) \left[ 1-\left( \chi +\sigma \right) \left( 1-\theta \right) \right] +\left( 1-\tau _{a}\right) \right\} }\nonumber \\&\quad \cdot \exp (-1)\nonumber \\&\quad \times \left\{ \frac{\left( 1-z_{x,L}+\Omega _{x,L}\cdot \iota \right) ^{1-\frac{1}{\alpha }}}{1-z_{r,L}+\Omega _{r,L}\cdot \iota }+\frac{\beta _{H}}{\beta _{L}}\cdot \frac{\varsigma _{L}}{\varsigma _{H}}\cdot \frac{\left( 1-z_{x,H}+\Omega _{x,H}\cdot \iota \right) ^{1-\frac{1}{\alpha }}}{1-z_{r,H}+\Omega _{r,H}\cdot \iota }\right. \nonumber \\&\quad \left. \cdot \frac{h}{l}\cdot \left( \frac{H}{L}\right) ^{1-\delta }\right\} \nonumber \\&\quad +\frac{\rho }{\left[ 1-\left( \chi +\sigma \right) \left( 1-\theta \right) \right] \cdot \left\{ \left( q^{\frac{1-\alpha }{\alpha }}-1\right) \left[ 1-\left( \chi +\sigma \right) \left( 1-\theta \right) \right] +\left( 1-\tau _{a}\right) \right\} }\nonumber \\&\quad -\frac{-\rho }{1-\left( \chi +\sigma \right) \left( 1-\theta \right) }\text {.} \end{aligned}$$

And notice that the sign of $\frac{\partial g^{*}}{\partial \iota }$ is determined by the expression inside the brackets, and that

$$\begin{aligned} \frac{\partial }{\partial \iota }\left[ \frac{\left( 1-z_{x,S}+\Omega _{x,S}\cdot \iota \right) ^{1-\frac{1}{\alpha }}}{1-z_{r,S}+\Omega _{r,S}\cdot \iota }\right]&=-\left( \frac{1-\alpha }{\alpha }\right) \Omega _{x,S}\frac{\left( 1-z_{x,S}+\Omega _{x,S}\cdot \iota \right) ^{-\frac{1}{\alpha }}}{1-z_{r,S}+\Omega _{r,S}\cdot \iota }\\&\quad -\Omega _{r,S}\frac{\left( 1-z_{x,S}+\Omega _{x,S}\cdot \iota \right) ^{1-\frac{1}{\alpha }}}{\left( 1-z_{r,S}+\Omega _{r,S}\cdot \iota \right) ^{2}}\\&<0,\quad \forall \,S\,\in \left\{ L,H\right\} , \end{aligned}$$

which implies $\frac{\partial g^{*}}{\partial \iota }<0$ and $\frac{\partial \pi ^{*}}{\partial \iota }>0$.

## Footnotes

1 For example, shortages of pharmaceuticals, food and other essential grocery items began with panic shopping due to possible shortages (Thompson 2020).

2 Alternatively, one might consider that most skilled workers operate in the public sector—health, education, justice, and government services—and the current pandemic has had a more severe impact on less skilled factory workers (e.g., Kikuchi et al. 2020).

3 Public sector workers have job protection and, on average, are paid about 11% more than their private sector counterparts, which is the average value in empirical data for the US and European Union Member States over the past two decades.

4 The average figure in empirical data for the US and EU Member States over the past two decades for long-term employment in the public and private sectors shows that public sector workers represent about 25% of all workers.

5 In the baseline case, we abstract from the more conventional CIA constraint on consumption, or, more generically, from money-in-utility or a liquidity/pecuniary-transaction-costs specification in the households’ optimization problem (e.g. Feenstra 1986), as we first wish to focus on the worldwide production and technology sectors of the model and their interaction with the monetary sector. Then, noting that the monetary policy’s effects can be magnified, we introduce, in Sect. 5, these assumptions as an extension to the baseline model.

6 In this paper, when we mention “output” we are more precisely referring to the “value added” of the economy. Since output is produced with labor and capital and, in our case, as it will be made clear further ahead, the factors are labor and intermediate goods, we can interpret intermediate goods in the context of our model as replacing the role of capital in the production function.

7 By real we mean that is the ratio of the price of the output in each sector to the price of the aggregate output. All prices and costs in this paper are to be regarded in this way.

8 Our production function is a Cobb-Douglas production function with equal shares of inputs and an infinite number of tasks indexed between 0 and 1. The production function takes this form if we analyze the discrete version of this one, in which $Y=Y_{1}^{1/n}\cdot Y_{2}^{1/n}\cdot ....\cdot Y_{n}^{1/n}$. Assuming perfect competition in both tasks and factor markets from the maximization problem of profits, which are given by $\Pi =Y-\sum _{i=1}^{n}p_{i}Y_{i}$, results that $p_{i}Y_{i}=\frac{Y}{n}$, which is constant. From here we can express the output as $Y=\sum _{i=1}^{n}p_{i}Y_{i}$, which becomes $Y=\int _{0}^{1}p_{n}Y_{n}\textrm{d}n$ as the number of tasks tend to infinite and if they are indexed between 0 and 1.

9 Hence, the monopolistic mark-up increases with $z_{x}$ and decreases with $\Omega _{x}$. We use to parametrize the intensity of the subsidy $z_{x}$ and of the CIA constraint on manufacturing of intermediate goods, $\Omega _{x}$, and we will be able to compare these parameters with the strength of the subsidy and of the CIA constraint on R &D, to be introduced below.

10 As in Afonso and Longras (2022), for example, the positive learning effect, (ii), is thus modeled in such a way that, together with the adverse effect, (iii), it totally offsets the positive influence of the quality rung on the profits of each intermediate leader firm, as we can see below.

11 The Frisch elasticity of labor supply is a useful indicator that gives the percentage increase in labor supply resulting from a 1% increase in the wage rate, while maintaining the marginal utility of wealth constant.

12 The value of these firms, in turn, corresponds to the value of patents in use.

13 It can be understood as a corporate tax.

14 $\tau _{w,L}$ and $\tau _{w,H}$ are the labor taxes, which, strictly speaking, also include contributions to social security and that in most advanced countries is around 40%. As we will see below, these taxes do not affect the economic growth rate; if there were human-capital accumulation, these taxes would penalize the respective accumulation.

15 From now on we will consider that $\tau _{w,H}$ equals $\tau _{w,L}$, freezing this fiscal policy channel to promote wage equality between sectors, although we will continue to consider the difference in these parameters in the expressions throughout the paper.

16 The need for a balanced budget imposes limits on tax policy action.

17 Remember that $z_{r}$ decreases the cost of R &D thus promoting R &D activity, while $z_{x}$ increases the monopolistic mark-up and, thereby, profits, thus acting as an incentive to R &D activities.

18 In addition to Chu and Cozzi (2014), some more recent studies along these lines are, for example, Chu and Ji (2016), Chu et al. (2017, 2019).

19 It will be shown below that the growth rate of the real money supply/balances is determined by the real growth rate of the economy in the steady-state equilibrium.

20 Indeed, $Y=Y_{L}+Y_{H}$, where $Y_{L}=l\cdot L\left[ \frac{P_{L}\cdot \left( 1-\alpha \right) }{q\left( 1-z_{x,L}+\Omega _{x,L}\cdot i\right) }\right] ^{\frac{1-\alpha }{\alpha }}Q_{L}$ and $Y_{H}=h\cdot H\left[ \frac{P_{H}\cdot \left( 1-\alpha \right) }{q\left( 1-z_{x,H}+\Omega _{x,H}\cdot i\right) }\right] ^{\frac{1-\alpha }{\alpha }}Q_{H}$.

21 Hence, the R &D cost decreases with $z_{r}$ and increases with $\Omega _{r}$.

22 We solve the model numerically because the differential equation describing the *G*’s path is nonlinear and because we want to look at the path of adjustment of our variables of interest.

23 In Appendix B it is shown that the model is indeed consistent, ensuring that is a dynamic general-equilibrium endogenous growth model, in which the aggregate final good, *Y*, is used in consumption, *C*, and investment, $X+R$. Moreover, firms and households are rational and solve their problems, free-entry R &D conditions are met, and markets clear.

24 As recent empirical evidence emphasizes a sustained decrease in labor force participation, particularly in the industrial sector (e.g., Elsby et al. 2013; Karabarbounis and Neiman 2014; Oberfield and Raval 2014), the expected results in this regard go in the direction of promoting growth and welfare and reducing inflation.

25 The more patient—i.e., the smaller the value of $\rho$—and the less keen the individuals are on consumption smoothing—i.e., the smaller the value of $\theta$—the higher the steady state growth rate.

26 An increase in $\iota$, by raising both the cost of intermediate-good production and the cost of R &D (whatever their relative magnitude), always reduces the incentive to allocate resources to growth-enhancing activities.

27 The public sector is more skilled intensive and, in addition, is guaranteed by the government.

28 In the standard DTC literature, the chain of effects is dominated by the market-size channel, by which technologies that use the more abundant type of labor are favored—i.e., interpret the rise in the skill premium as a result of the market-size effect. Building on this literature, we analyze the direction of technological knowledge in a dynamic setting where, in line with the dominant literature on scale effects since Jones (1995), the scale effects are strongly removed and the chain of effects is deeply induced by the price channel, by which there are stronger incentives to improve technologies when the goods that they produce command higher prices, i.e., technologies that use the scarcer labor are favored.

29 In doing so, the usual approach in the literature is to consider that the (static) CIA constraint is binding; that is $b(t)=m(t)$.
